# The evolution of artificial intelligence technology in non-alcoholic fatty liver disease

**DOI:** 10.3389/fradi.2025.1634165

**Published:** 2025-09-16

**Authors:** Jiawen He, Yao Wu, Zhiyong Lin, Ruohong He, Li Zhuo, Yingying Li

**Affiliations:** ^1^ School of Nursing, Guangdong Pharmaceutical University, Guangzhou, Guangdong, China; ^2^ Guangdong Medical University, Zhanjiang, Guangdong, China; ^3^ Guangdong Geriatrics Institute, Guangdong Provincial People’s Hospital (Guangdong Academy of Medical Sciences), Southern Medical University, Guangzhou, China

**Keywords:** artificial intelligence, non-alcoholic fatty liver disease, metabolic-associated fatty liver disease, metabolic dysfunction-associated steatotic liver disease, bibliometric analysis, hepatology, liver disease

## Abstract

**Background:**

The incidence of Non-alcoholic Fatty Liver Disease (NAFLD) continues to rise, becoming one of the major causes of chronic liver disease globally and posing significant challenges to healthcare systems worldwide. Artificial intelligence (AI) technology, as an emerging tool, is gradually being integrated into clinical practice for NAFLD, providing innovative approaches to improve diagnostic efficiency, personalized treatment plans, and disease prognosis assessment. However, current research remains fragmented, lacking systematic and comprehensive analysis.

**Objective:**

This study conducts a bibliometric analysis of artificial intelligence applications in Non-alcoholic Fatty Liver Disease (NAFLD), aiming to identify research trends, highlight key areas, and provide comprehensive and objective insights into the current state of research in this field. We expect that these research results will provide valuable references for guiding further research directions and promoting the effective application of AI in liver disease healthcare.

**Methods:**

This study used the Web of Science Core Collection database as the data source, searching the Science Citation Index Expanded (SCI-Expanded) and Current Chemical Reactions (CCR-Expanded) citation indexes. The search timeframe was set to include all relevant literature from 2010 to March 25, 2025. The research methodology adopted a multi-software joint analysis strategy: First, HistCite Pro 2.1 was used to analyze the historical evolution and citation relationships of literature in this field. The tables generated by the tool systematically recorded the development process of the literature, clearly depicting the evolution of the research field over time. Second, Scimago Graphica was used to create a country/region collaboration network view, intuitively showing academic collaboration among countries/regions (SCImago Lab, 2022). VOSviewer 1.6.20 was used to analyze collaboration networks and visualize keyword co-occurrences to identify main research themes and clusters. CiteSpace was used for deeper scientific literature analysis, precisely capturing the dynamic changes of research hotspots and the evolution of frontier trends through Burst Detection algorithms and Timezone View.

**Results:**

A total of 655 papers were retrieved from 60 countries, 1462 research institutions, and 4,744 authors published in 279 journals. The number of papers surged dramatically during 2019–2024, with papers from these six years accounting for approximately 83.8% (549/655) of the total. Country-level analysis showed that the United States and China are the major contributors to this field; journal analysis indicated that Scientific Reports and Diagnostics are the journals with the highest publication volumes. In-depth analysis of 655 publications revealed four major research clusters: non-invasive assessment methods for liver fibrosis, imaging-based diagnosis (magnetic resonance imaging, CT, and ultrasound), disease progression prediction model construction, and biomarker screening genes. Recent research trends indicate that deep learning algorithms and multimodal data fusion have become research hotspots in AI applications for NAFLD diagnosis and treatment. Particularly, MRI-based liver fat quantification and fibrosis assessment, combined with deep learning technologies for non-invasive diagnostic methods, show potential to replace liver biopsy.

**Conclusion:**

This study comprehensively outlines the development trajectory and knowledge structure of artificial intelligence technology in NAFLD research through systematic bibliometric analysis. The findings suggest that although the field faces challenges such as data standardization and model interpretability, AI technology shows broad prospects in NAFLD disease management and risk prediction. Future research should focus on multimodal data fusion, clinical translation, and evaluation of practical application value to promote the realization of AI-assisted precision medicine for NAFLD. This study not only depicts the current landscape of artificial intelligence applications in NAFLD but also provides a reference basis for future development in this field.

## 1 Introduction

Non-alcoholic fatty liver disease (NAFLD) represents one of the most prevalent chronic liver disorders globally (1–4), with worldwide prevalence reaching 30.05% and demonstrating a remarkable 50.4% increase from 1990 to 2019 (5). The geographic distribution exhibits substantial heterogeneity, ranging from 44.37% in Latin America to 25.10% in Western Europe. NAFLD serves not only as a principal etiologic factor for liver cirrhosis and hepatocellular carcinoma (6) but also demonstrates significant associations with multiple metabolic disorders, including type 2 diabetes mellitus and cardiovascular diseases (7). The recent nomenclature evolution from NAFLD to MASLD (Metabolic dysfunction-Associated Steatotic Liver Disease) (8, 9) has created novel opportunities for artificial intelligence (AI) technology implementation. In contrast to conventional NAFLD definitions, MASLD emphasizes quantitative assessment of metabolic dysfunction, which demonstrates high concordance with AI technology's inherent advantages in multidimensional data integration and pattern recognition (10). Given that this study encompasses a historical timeframe spanning from 2010 to 2025, during which NAFLD terminology maintained predominance, we uniformly adopt NAFLD as the primary nomenclature for analysis while incorporating all relevant concepts in our search strategy to ensure comprehensive coverage.

**Figure 1:**
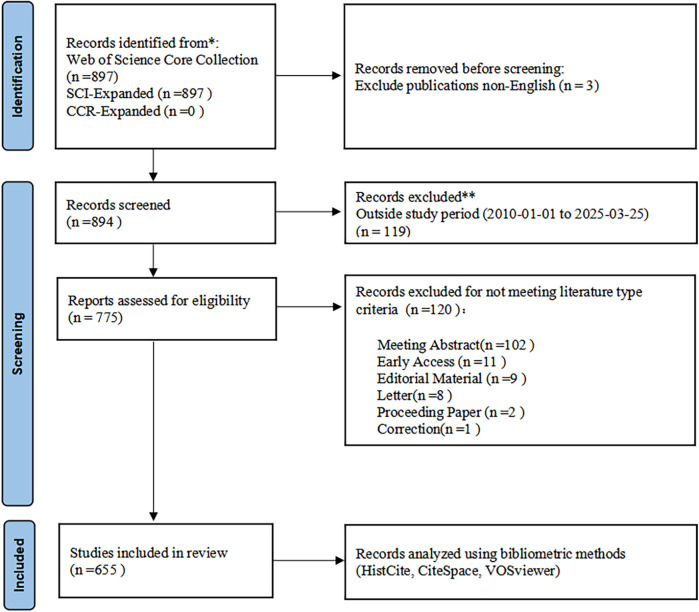
Modified PRISMA flow diagram incorporating bibliometric analysis methods.

Traditional diagnostic approaches for NAFLD primarily rely on liver biopsy (11), which, despite providing accurate histopathological information, presents inherent invasiveness limitations. Although imaging modalities offer non-invasive alternatives, they remain constrained by operator dependency and insufficient sensitivity for early-stage disease detection. The NAFLD disease spectrum encompasses a complex pathological progression from simple steatosis (NAFL) to liver cirrhosis and hepatocellular carcinoma (HCC), presenting challenges for accurate prediction and monitoring through conventional methodologies (12). These inherent limitations have prompted researchers to pursue more efficient and precise diagnostic and management approaches.

The rapid development of artificial intelligence (AI) technology has brought revolutionary opportunities for NAFLD diagnosis and treatment. AI is an interdisciplinary field that refers to using computers to simulate human intelligent behavior and train computers to learn human behavior (13). AI includes various technologies such as machine learning (ML), deep learning (DL), robotics, and natural language processing (NLP), which are rapidly developing and playing indispensable roles in social development, particularly in medical applications (14).

In NAFLD research, AI demonstrates unique advantages: DL can screen disease characteristic genes (15) to determine potential therapeutic targets, thereby achieving the purposes of prediction, prevention, and personalized treatment; ML can be used to detect fibrosis and NASH (16). Artificial intelligence can integrate multi-source data, including clinical indicators, biochemical examinations, imaging features, pathological images, and even genomic data, potentially providing more precise NAFLD diagnosis and risk stratification tools (17).

More critically, contemporary literature lacks comprehensive analysis of academic networks and collaborative frameworks within this field, including identification of principal contributing nations and institutions, characterization of inter-research team collaborative relationships, and analysis of regional variations in technological pathway selections. Furthermore, existing literature reviews predominantly employ qualitative analytical methodologies, which, while capable of summarizing specific technological application statuses, cannot quantitatively capture the dynamic evolution patterns of research hotspots or predict future developmental trends based on large-scale bibliometric evidence. The methodological innovations spanning from early traditional statistical approaches to machine learning algorithms, and subsequently to recent deep learning and multimodal data fusion technologies, lack precise quantitative characterization regarding their temporal nodes, driving factors, and impact scope.

Bibliometric analysis serves as an effective tool for evaluating and tracking the development of specific research fields (18). By applying mathematical and statistical methods to analyze published literature, this approach can identify research hotspots, collaboration networks, and knowledge foundations, thereby revealing the developmental trajectory of academic disciplines (19). Through analyzing annual publication trends and citation patterns of AI research in NAFLD, we aim to: (1) assess the overall developmental dynamics of this field; (2) identify major contributing countries/regions, research institutions, and core authors to reveal global research distribution patterns and collaboration networks; (3) determine high-impact journals and key literature to establish a knowledge map of the field; (4) identify research hotspots, thematic clusters, and technological evolution pathways through keyword co-occurrence and burst analysis; (5) explore research progress in different AI application domains within NAFLD (such as diagnosis, risk stratification, and disease progression prediction); and (6) analyze current research limitations and challenges to provide recommendations for future research directions.

The innovation of this study lies in its systematic analysis of the comprehensive landscape and evolutionary trajectory of AI applications in NAFLD from a bibliometric perspective. Distinguished from previous systematic reviews that primarily focused on clinical application efficacy, this research employs a multi-software joint analysis strategy to reveal research directions, collaboration networks, and knowledge structures within this field. Rather than merely describing the current status of artificial intelligence applications in NAFLD, this study tracks the dynamic migration of research hotspots and evolution patterns of frontier trends, thereby providing directional guidance for future research endeavors.

## 2 Methods

### 2.1 Data sources and search strategy

This study utilized the Web of Science Core Collection (WoSCC) as the primary data source. Web of Science represents the world's most trusted and publisher-independent global citation database (20). The search timeframe was set from January 1, 2010, to March 25, 2025, to analyze developmental trends in this field.The search strategy was developed through multiple rounds of expert discussions and pre-search optimization. First, we identified NAFLD-related terms by combining Medical Subject Headings (MeSH) terms with free text words, including recently proposed concepts such as metabolic-associated fatty liver disease (MAFLD) and metabolic dysfunction-associated steatotic liver disease (MASLD) (8). Second, for the artificial intelligence domain, we incorporated not only broad terms such as “artificial intelligence” and “machine learning,” but also specifically included specialized techniques commonly used in medical imaging analysis, such as “convolutional neural network” and “radiomics,” to ensure comprehensive retrieval.The search formula was constructed using Boolean logical operators (AND and OR). To ensure the effectiveness and comprehensiveness of the search strategy, we employed a three-fold validation method: (1) Recall validation: We conducted retrospective testing using 10 high-impact representative articles in this field as benchmarks, achieving 100% recall rate with our final search strategy; (2) Precision assessment: We randomly sampled 200 articles from preliminary search results for manual relevance evaluation, achieving 89.5% relevance ratio; (3) Iterative optimization: We balanced search sensitivity and specificity through multiple optimization rounds.The validated and optimized final search strategy is as follows:

TS = (“non-alcoholic fatty liver disease” OR “NAFLD” OR “MAFLD” OR “metabolic associated fatty liver disease” OR “MASLD” OR “metabolic dysfunction associated steatotic liver disease” OR “steatotic liver disease” OR “non-alcoholic steatohepatitis” OR “hepatic steatosis” OR “Liver Fat”) AND (“artificial intelligence” OR “machine learning” OR “deep learning” OR “decision making tree” OR “random forest” OR “support vector machines” OR “radiomics” OR “convolutional neural network” OR “clinical decision support system*” OR “predictive model” OR “medical image analysis”)

The selection of the Web of Science Core Collection as the sole data source was based on the methodological characteristics and data quality requirements of bibliometric research. Unlike systematic reviews that pursue absolute comprehensiveness in literature collection, bibliometric analysis prioritizes data standardization and citation network integrity, which are crucial for accurate co-citation analysis, burst detection, and other core analytical procedures.

### 2.2 Literature screening

This study adhered to the transparency principles of the PRISMA 2020 guidelines (21) during the literature identification and screening phases to ensure the systematicity and completeness of data used for bibliometric analysis (Figure 1).

#### 2.2.1 Inclusion criteria

To ensure research quality, we established the following inclusion criteria:

a. Research articles focusing on artificial intelligence technologies in non-alcoholic fatty liver disease (NAFLD), with no restrictions on study design
b. Articles published in journals indexed in WoSCC
c. For duplicate publications, the earliest published and most complete version was included
d. Article types limited to original research articles or review articles
e. Only articles published in English were included

#### 2.2.2 Exclusion criteria

a. Conference abstracts, news reports, commentaries, letters, and retracted publications
b. Articles with only abstracts available and without full text
c. Articles irrelevant to the research topic

Following the preliminary search, two researchers independently reviewed all retrieved literature by examining titles and abstracts according to the aforementioned criteria to ensure all included articles were relevant to the research topic. In cases of disagreement, we employed a quantitative assessment approach: first recording the types and frequency of disagreements, then reaching consensus through discussion. When disagreements persisted, a third researcher was consulted for final determination.

### 2.3 Data analysis and visualization tools

The theoretical foundation for employing a multi-software integrated analysis approach in this study stems from the limitation that individual tools cannot comprehensively address the multifaceted dimensions inherent in bibliometric investigations.

HistCite Pro 2.1, developed by Eugene Garfield, the originator of the Science Citation Index (SCI), was utilized to examine the historical development and citation patterns within this research domain. This software demonstrates distinctive capabilities in citation network processing and core literature identification (22). The analytical framework employs two fundamental metrics: Total Local Citation Score (TLCS) and Total Global Citation Score (TGCS). TLCS quantifies the frequency with which a specific publication is cited by other works within the current dataset, thereby indicating the academic influence of the publication within the designated research field. TGCS measures the aggregate citation frequency of the publication within the Web of Science database, representing its comprehensive impact across the global academic community. The concurrent application of these metrics facilitates a thorough assessment of both academic merit and influence scope.Consequently, HistCite functions as a robust citation analytical instrument, demonstrating efficacy in identifying the most influential authors, journals, and publications (23).

Scimago Graphica was employed to generate country/region collaboration network visualizations, providing intuitive representation of inter-regional academic collaboration patterns (SCImago Lab, 2022).

VOSviewer, a Java-based visualization platform developed by the Centre for Science and Technology Studies at Leiden University, demonstrates particular suitability for processing large-scale bibliometric datasets and generating diverse network analyses, encompassing co-occurrence networks, citation networks, and term frequency analyses (24). In the present investigation, VOSviewer 1.6.20 was implemented for collaboration network analysis and keyword co-occurrence visualization to identify principal research themes and cluster formations. The keyword co-occurrence network analysis employed standardized parameters including: analytical unit specification (author keywords selection), enumeration methodology (full counting implementation), and minimum threshold criteria (minimum keyword occurrence frequency = 4). Prior to keyword analysis, manual standardization of semantically equivalent keywords was conducted by the research team. This standardization procedure was executed independently by two investigators through systematic keyword inventory examination, identification of semantically similar terms, and establishment of a standardized lexicon, with discrepancies resolved through consensus discussion.Standardization examples include the unification of “Non-Alcoholic Fatty Liver Disease” and “Nonalcoholic Fatty Liver Disease” as “NAFLD,” and the consolidation of “metabolism” and “metabolomics” under “metabolomics.”

CiteSpace v.6.4.R1, developed by Chaomei Chen, was employed for comprehensive scientific literature analysis (25). Through its distinctive burst detection algorithm and timeline visualization, temporal analysis of keywords and citations was conducted to precisely capture the dynamic evolution of research hotspots and emerging trend development. The time-slicing parameter was configured to one-year intervals to ensure precision and continuity in research evolution trends. For keyword burst detection, *γ* was set to 0.7 to capture early signals of emerging research concepts; for citation burst detection, *γ* was established at 1.0 to identify milestone studies with sustained academic impact. Node selection criteria utilized the g-index algorithm with k-value set to 25 for each time slice, ensuring network representativeness while controlling complexity.Network pruning employed the Pathfinder algorithm combined with pruning sliced networks strategy to effectively simplify network structure while preserving critical pathway information.

Additionally, SPSS 26.0 software was utilized for time series regression analysis to quantitatively assess developmental trends of AI in NAFLD research. Through construction of a log-linear regression model, publication volume variation patterns over time were analyzed, with the natural logarithm of publication count as the dependent variable and standardized temporal coding as the independent variable. Model specifications included residual normality testing, heteroscedasticity detection, and collinearity diagnostics to ensure statistical inference validity. Regression analysis employed the enter method with significance level set at *α* = 0.05.

This multi-software complementary analytical strategy not only enhanced research comprehensiveness and reliability but also achieved multi-level visualization analysis spanning from macroscopic to microscopic perspectives and from static to dynamic dimensions.

## 3 Results

### 3.1 Analysis of annual publication volume and average citation frequency

**Figure 2:**
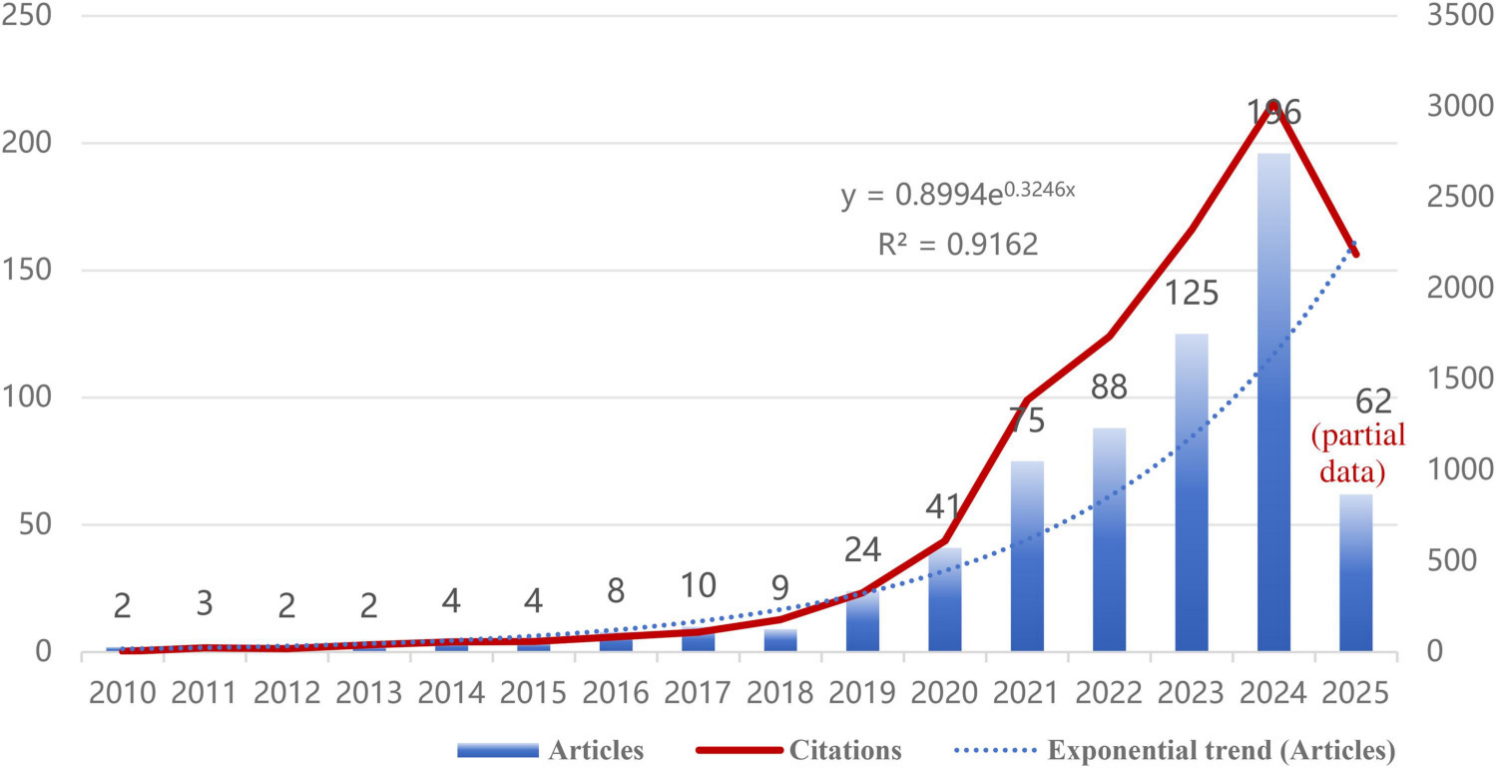
Annual publication volume and average citation frequency trends for artificial intelligence in non-alcoholic fatty liver disease (NAFLD) research from 2010 to 2025.

This study included 655 papers on artificial intelligence applications in non-alcoholic fatty liver disease (NAFLD). As shown in Figure 2, AI development in NAFLD research can be clearly divided into three stages: embryonic period (2010–2018), rapid growth period (2019–2022), and explosive period (2023–2024). Exponential regression analysis demonstrated R^2^ = 0.9162 (*p* *<* *0.001*), indicating a significant exponential growth trajectory in this field. Since 2010, the publication volume in this field has shown steady growth, gradually increasing from 2 papers annually to 196 papers in 2024. Particularly during 2019–2024, the number of papers surged, with publications from this five-year period accounting for approximately 83.8% (549/655) of the total. Although the publication volume in 2025 decreased to 62 papers, this reduction may be attributed to incomplete data collection for that year, with certain articles potentially unindexed during the retrieval process.

### 3.2 Analysis of countries/regions and institutions

Based on the Total Local Citation Score (TLCS, representing citations within the current dataset) and Total Global Citation Score (TGCS, representing total citations in the Web of Science database) derived from HistCite analysis, we conducted an in-depth examination of the international distribution patterns in NAFLD artificial intelligence application research.

**Figure 3:**
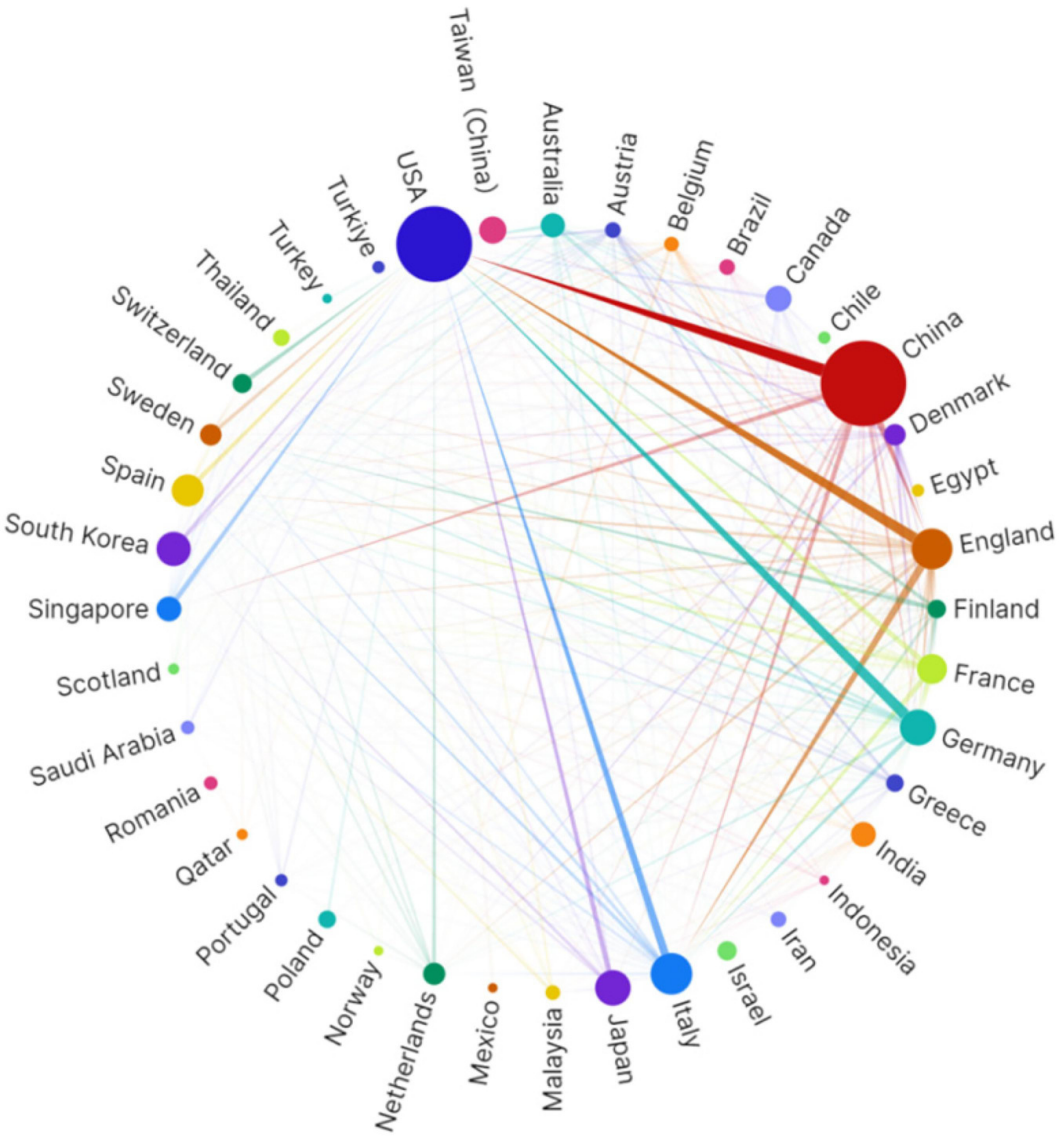
Visualization of countries/regions collaboration network generated based on scimago graphical.

China and the United States demonstrated outstanding performance in this field (Table 1). Notably, although China leads in publication volume (237 articles, 36.2%), the United States exhibits significantly higher local citation scores (TLCS = 376) and global citation scores (TGCS = 6,751) compared to China, reflecting potentially greater academic impact of American research. European countries (Italy, the United Kingdom, Germany, Spain, and France) collectively contributed approximately 32.8% of research outputs, constituting the second-largest research cluster in this field. Italy and the United Kingdom tied for third place, each publishing 56 papers (8.5%). Particularly noteworthy is the United Kingdom, which, despite having the same publication volume as Italy, achieved markedly higher citation impact with TLCS = 127 and TGCS = 2,090, reflecting the quality advantage of British research. Beyond China and Taiwan, the Asian region also includes South Korea and Japan among the top contributors, demonstrating widespread Asian participation in this field. These international distribution characteristics not only reflect each country's research strength and attention in the intersection of NAFLD and artificial intelligence research but also indicate potential directions for future international collaboration in this domain. This pattern is also reflected in the institutional publication rankings shown in Table 2A. It is worth noting that some countries, such as France, despite relatively modest publication volumes (29 articles, 4.4%), achieved relatively high citation scores. The network diagram in Figure 3 illustrates collaborative relationships among countries, with line thickness representing collaboration intensity. The thickest connection between the United States and China indicates the closest collaborative relationship between these two nations in this field. The United Kingdom serves as the third major node, maintaining extensive connections with multiple countries.

**Table 1:** Top 10 countries/regions by number of publications.

Rank	Country	Publications, *n* (%)	TLCS^a^	TGCS^b^
1	China	237 (36.2%)	177	2,855
2	USA	186 (28.4%)	376	6,751
3	Italy	56 (8.5%)	47	991
4	UK	56 (8.5%)	127	2,090
5	Germany	42 (6.4%)	38	1,015
6	Japan	41 (6.3%)	36	778
7	South Korea	38 (5.8%)	11	427
8	Spain	33 (5.0%)	30	840
9	France	29 (4.4%)	74	1,918
10	Taiwan(China)	24 (3.7%)	32	395

TLCS^a^, Total Local Citation Score, reflecting the influence of literature within the specific collection of literature currently being analyzed.

TGCS^b^, Total Global Citation Score, reflecting the influence of literature in the broader global academic community.

Through the institutional publication ranking data (Table 2A), we found that the University of California San Diego leads with 24 publications (3.7%). Notably, the Chinese University of Hong Kong achieved a remarkably high Total Global Citation Score (TGCS) of 1,261 despite only 16 publications, demonstrating substantial global academic impact. The University of Wisconsin-Madison recorded a Total Local Citation Score (TLCS) of 128, showing significant influence in specific domains, particularly in CT imaging quantitative analysis.From the collaboration network activity perspective (Table 2B), Wenzhou Medical University ranks first with a total link strength of 72, reflecting its pivotal role in international collaboration networks, followed by the Chinese University of Hong Kong (55) and the University of Verona (43). This demonstrates the prominent performance of Chinese institutions in NAFLD-AI research collaboration networks.

**Table 2A:** Top 10 institutions by number of publications(including ties, 12 institutions total).

Rank	Institution	Country	Publications, *n* (%)	TLCS^a^	TGCS^b^
1	University of California, San Diego	United States	24 (3.7%)	70	1,852
2	Capital Medical University	China	22 (3.4%)	28	149
3	University of Wisconsin-Madison	United States	18 (2.7%)	128	927
4	Wenzhou Medical University	China	17 (2.6%)	45	762
5	The Chinese University of Hong Kong	China	16 (2.4%)	85	1,261
6	Zhejiang University	China	15 (2.3%)	18	178
7	Kurume University	Japan	13 (2.0%)	3	284
8	Virginia Commonwealth University	United States	13 (2.0%)	41	433
9	Harbin University	China	12 (1.8%)	1	30
10	Harvard Medical School	United States	12 (1.8%)	11	312
11	University of Oxford	UK	12 (1.8%)	53	838
12	University of Verona	Italy	12 (1.8%)	13	146

TLCS^a^, total local citation score. TGCS^b^, total global citation score.

**Table 2B:** Top 10 institutions by total link strength in collaboration networks.

Rank	Institution	Countries	TLS^a^
1	Wenzhou Medical University	China	72
2	Chinese University of Hong Kong	China	55
3	University of Verona	Italy	43
4	Virginia Commonwealth University	United States	42
5	University Hospital Southampton	UK	42
6	National University of Singapore	Singapore	36
7	Hangzhou Normal University	China	32
8	University of Southampton	Singapore	32
9	Key Laboratory of Diagnosis and Treatment Development of Chronic Liver Disease, Zhejiang Province	China	31
10	Capital Medical University	China	30

TLS^a^, total link strength.

**Figure 4:**
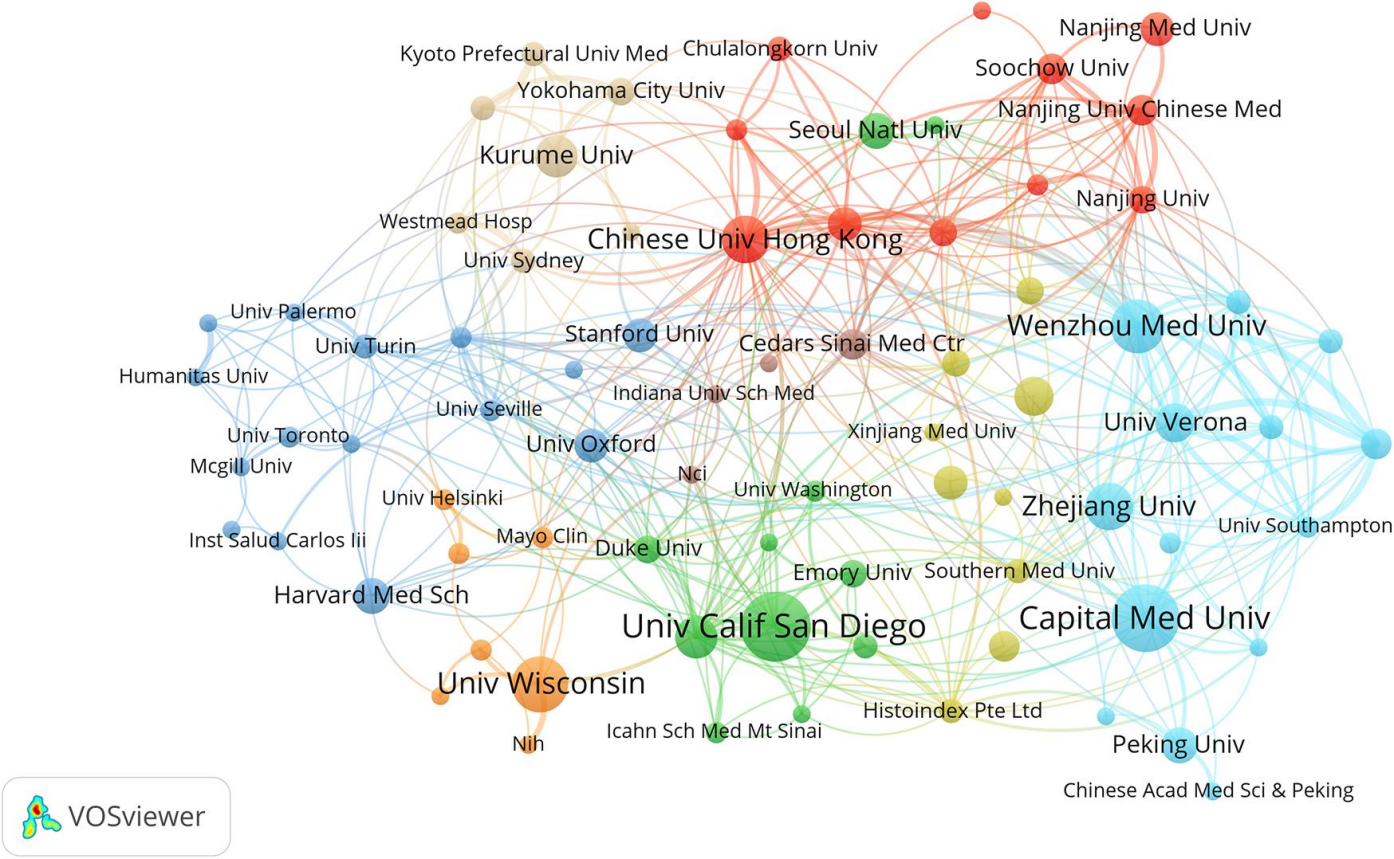
Visualization of research networks of institutions. Institutional collaboration network clustering analysis of artificial intelligence in non-alcoholic fatty liver disease research. Different colors represent different research clusters: the red cluster is centered around The Chinese University of Hong Kong, the green cluster is centered around University of California San Diego, and the blue cluster is represented by Capital Medical University, Zhejiang University, and Wenzhou Medical University. Node size indicates institutional publication volume/influence, while connecting lines represent collaboration relationship strength. This demonstrates the international collaboration network and major research communities in the application field of artificial intelligence in NAFLD research.

Figure 4 presents the institutional collaboration network cluster analysis, clearly revealing three major research clusters: the US cluster centered on the University of California San Diego; the Hong Kong cluster centered on the Chinese University of Hong Kong; and the mainland China research network cluster represented by Capital Medical University and Zhejiang University.

The analysis indicates that the Chinese University of Hong Kong possesses significant advantages in AI medical imaging analysis. Institutions such as Harbin Medical University and Nanjing Medical University have relatively recent publication timelines, particularly during 2023–2024 (Supplementary Figure S1), suggesting that mainland China's NAFLD artificial intelligence research is exhibiting a “late-mover advantage” trajectory.

### 3.3 Author and co-authorship network analysis

A total of 4,744 authors participated in the publications included in the analysis. Table 3 presents the top ten authors ranked by publication count. Rohit Loomba emerged as the most prolific author in this field with 17 papers (2.6%), followed by Perry J. Pickhardt, Vincent Wai-Sun Wong, and Ming-Hua Zheng. Vincent Wai-Sun Wong achieved a leading TGCS of 1,156, indicating significant research impact.

**Table 3:** Top 10 authors who published research papers.

Rank	Author	Institution	Country	Publications, *n* (%)	TLCS	TGCS
1	Rohit Loomba	University of California, San Diego	United States	17 (2.6%)	61	1,768
2	Perry J. Pickhardt	University of Wisconsin	United States	16 (2.4%)	96	843
3	Vincent Wai-Sun Wong	The Chinese University of Hong Kong	China	14 (2.1%)	84	1,156
4	Ming-Hua Zheng	Zhejiang University	China	14 (2.1%)	45	747
5	Takumi Kawaguchi	Kurume University School of Medicine	Japan	13 (2.0%)	3	284
6	Arun J. Sanyal	Virginia Commonwealth University	United States	11 (1.7%)	41	401
7	Ronald M. Summers	National Institutes of Health Clinical Center	United States	11 (1.7%)	76	601
8	Jia Li	Key Laboratory of Precision Diagnosis and Treatment	China	10 (1.5%)	1	40
9	Christopher D. Byrne	University of Southampton	United Kingdom	9 (1.4%)	15	123
10	Takeshi Okanoue	Saiseikai Suita Hospital	Japan	9 (1.4%)	23	157

**Figure 5:**
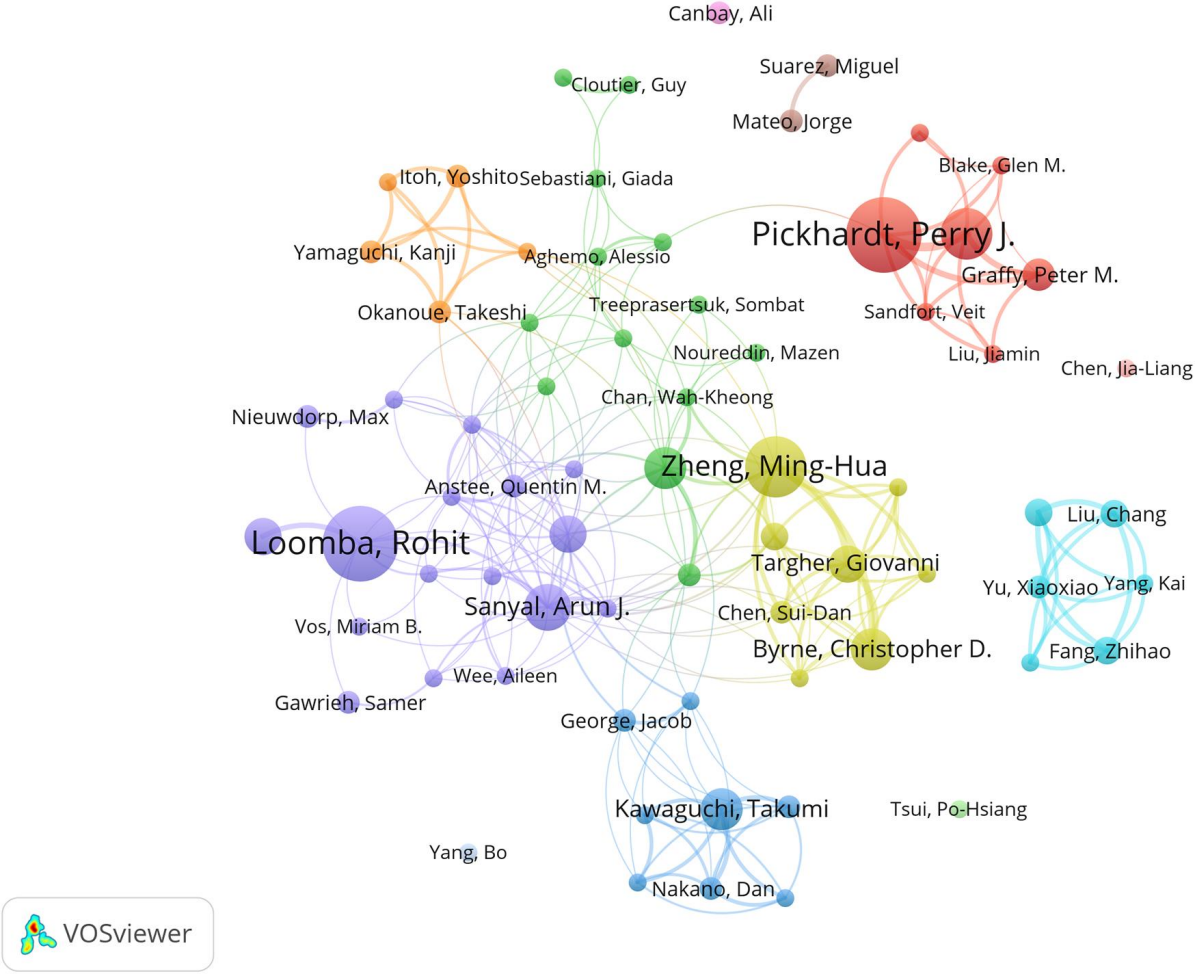
Visualization of research networks of authors.

The author collaboration network analysis in Figure 5 reveals three distinct clusters: the red cluster centered on Pickhardt, Perry J. (16 papers, TGCS = 843), primarily focusing on artificial intelligence applications in computed tomography; the blue cluster comprising Asia-Pacific researchers including Wong, Vincent Wai-Sun, specializing in clinical diagnostic algorithm development; and the green cluster representing European research alliance contributions to this field.

Additionally, through co-citation author network analysis (Supplementary Figures S2–S3), we identified Younossi as a core academic authority whose work likely established the foundation for NAFLD definition, epidemiological characteristics, and clinical significance. The David E. Kleiner cluster (163 co-citations) focuses on AI-assisted histopathological diagnosis, the Perry J. Pickhardt cluster (180 co-citations) leads deep learning algorithm development for medical imaging, the Paul Angulo cluster (130 co-citations) concentrates on clinical decision support system construction, while the Laurent Castera cluster advances intelligent applications of non-invasive diagnostic technologies (Supplementary Table S2).

### 3.4 Journal analysis

**Figure 6:**
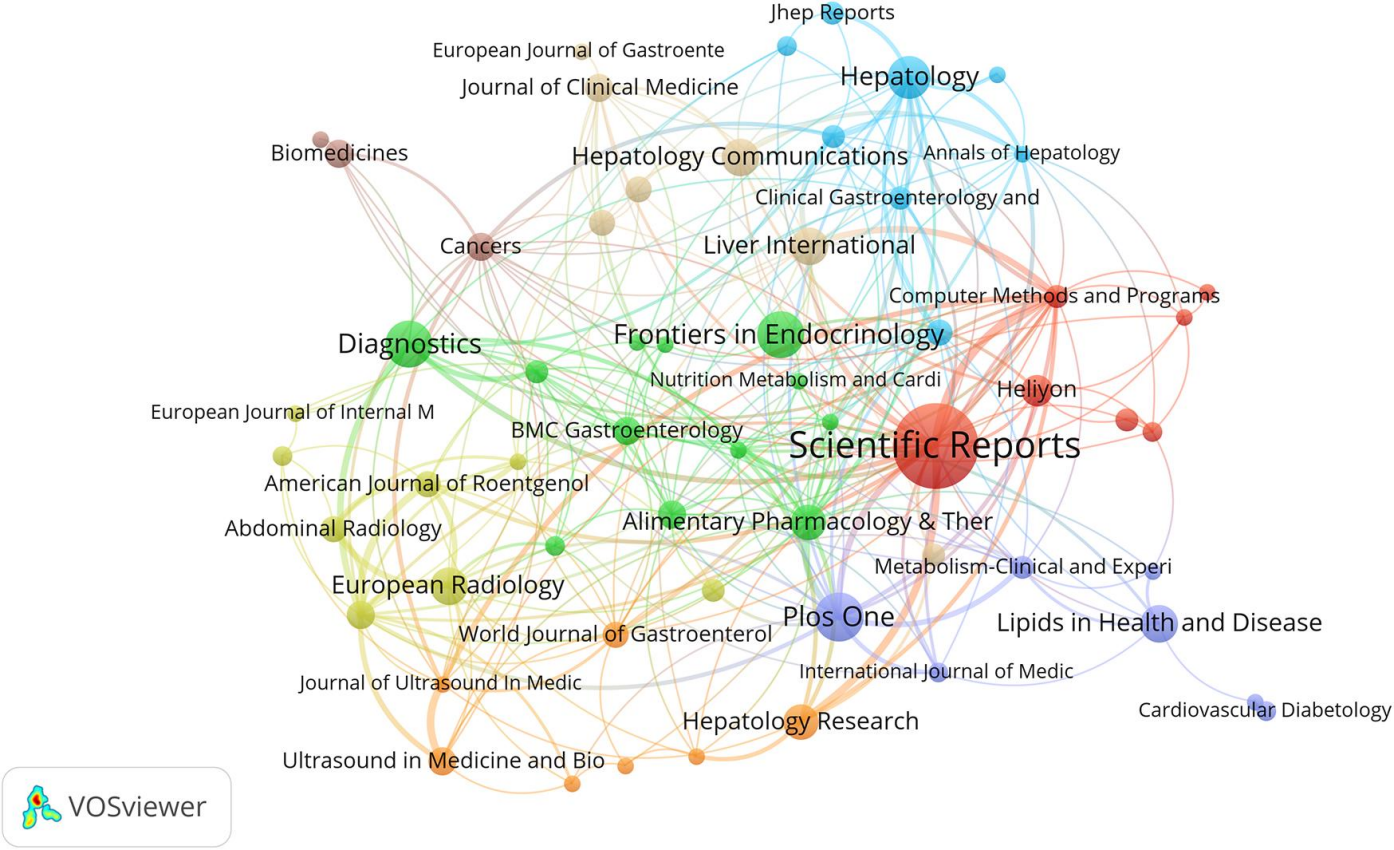
Visualization of research networks of journals.

Journal analysis plays an irreplaceable role in identifying core journals within specific fields. Table 4 lists the top ten journals ranked by publication count along with their respective impact factors (IF) and categories based on the Science Citation Index Expanded (SCIE). Scientific Reports achieved the highest publication volume (43 articles, 6.6%), while Hepatology demonstrated a high IF (2024) of 13.0 and ranked second in TGCS. In Figure 6, Scientific Reports appears as the large central node in the network, occupying a core position in this field. The figure also shows the presence of Frontiers series journals and other open-access publications.

**Table 4:** Top 10 journals by publication volume in artificial intelligence research on Non-alcoholic fatty liver disease (NAFLD) and their academic metrics.

Rank	Journal	Publications, *n* (%)	TLCS^a^	TGCS^b^	IF^c^ (2023)	Category (SCIE)
1	Scientific Reports	43 (6.6%)	0	849	3.9	MULTIDISCIPLINARY SCIENCES
2	PLoS ONE	17 (2.6%)	0	283	2.6	MULTIDISCIPLINARY SCIENCES
3	Diagnostics	16 (2.4%)	0	88	3.3	MEDICINE, GENERAL & INTERNAL
4	Frontiers in Endocrinology	16 (2.4%)	0	88	4.6	ENDOCRINOLOGY & METABOLISM
5	Hepatology	14 (2.1%)	38	588	13.0	GASTROENTEROLOGY & HEPATOLOGY
6	European Radiology	11 (1.7%)	17	118	4.7	RADIOLOGY, NUCLEAR MEDICINE & MEDICAL IMAGING
7	Hepatology Communications	11 (1.7%)	14	221	5.7	GASTROENTEROLOGY & HEPATOLOGY
8	Lipids in health and disease	11 (1.7%)	0	77	4.2	BIOCHEMISTRY & MOLECULAR BIOLOGY
9	Liver International	11 (1.7%)	11	349	5.2	GASTROENTEROLOGY & HEPATOLOGY
10	Alimentary Pharmacology & Therapeutics	10 (1.5%)	35	237	6.6	GASTROENTEROLOGY & HEPATOLOGY; PHARMACOLOGY & PHARMACY

TLCS^a^, Total Local Citation Score; TGCS^b^, Total Global Citation Score; IF^c^, Impact Factor.

Based on the journal publication temporal heat distribution map (Supplementary Figure S4), this visualization reveals the temporal distribution patterns of AI-NAFLD related research across different journals. Some journals such as Scientific Reports and PLoS One maintained high publication activity throughout the entire research period, while different journals exhibited distinct contribution characteristics across various time periods.

**Figure 7:**
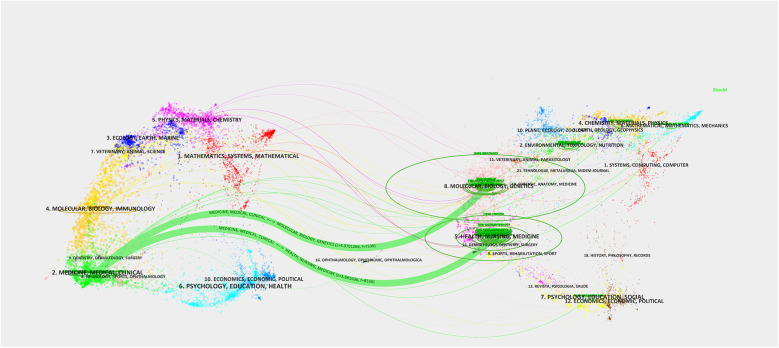
Journal dual graph overlay.

The journal dual-map overlay network provides important insights into understanding the interdisciplinary nature of AI-NAFLD research. Figure 7 uses colored node clusters to represent different disciplinary domains, with connections of varying thickness (particularly the thick green lines) illustrating the primary pathways and intensity of knowledge flow.

### 3.5 Keyword co-occurrence and keyword burst analysis

Co-occurrence analysis of keywords facilitates better understanding of research frontiers and developmental trends. The keyword ranking table (Table 5) reveals explosive growth in the integration of NAFLD research with AI technology, with NAFLD (231 occurrences, TLS = 476) and machine learning (163 occurrences, TLS = 370) occupying dominant positions. This high-frequency keyword co-occurrence indicates that AI technology has become the core methodological foundation for NAFLD research.

**Table 5:** Top 20 keywords by frequency of occurrence.

Rank	Keyword	Occurrences	TLS	Rank	Keyword	Occurrences	TLS
1	NAFLD	231	476	11	Biomarker	24	58
2	Machine Learning	143	319	12	Radiomics	20	49
3	Artificial Intelligence	70	179	13	Nomogram	18	45
4	Liver Steatosis	52	113	14	Liver	17	40
5	Liver Fibrosis	51	141	15	Diagnosis	15	43
6	Deep Learning	49	119	16	Metabolic Syndrome	15	40
7	Nash	34	98	17	Hepatocellular Carcinoma	14	32
8	Prediction Model	33	85	18	Masld	14	24
9	Fatty Liver Disease	32	84	19	Type 2 Diabetes	14	37
10	Non-Alcoholic Steatohepatitis	31	83	20	Magnetic Resonance Imaging	13	34

TLS, total link strength.

**Figure 8:**
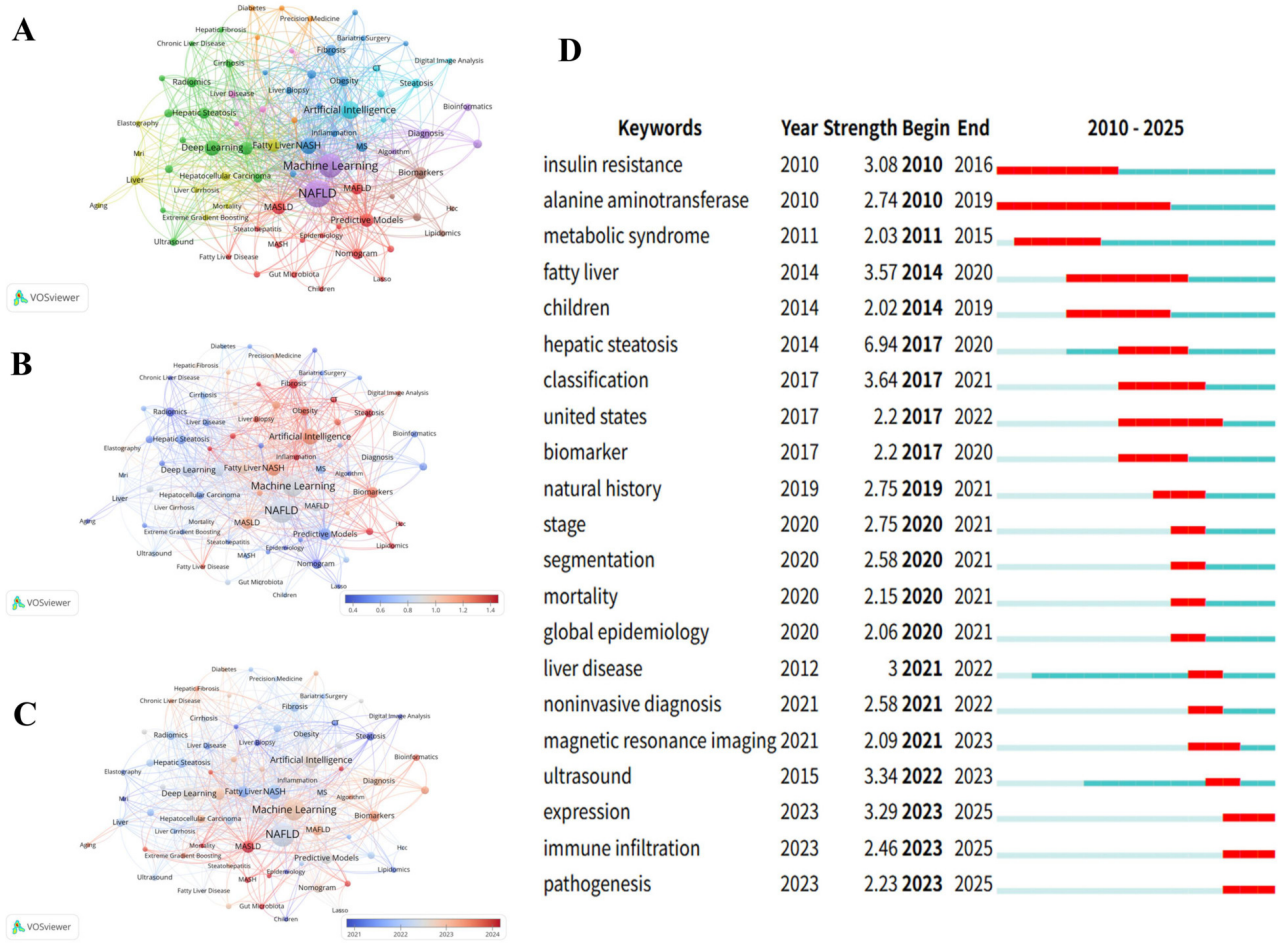
Keyword Co-occurrence network analysis: knowledge graph and evolution trends. **(A)**: NAFLD Research Topic Cluster Analysis.This figure identifies research direction clusters with different colors: Applications of Artificial Intelligence (AI) and Machine Learning (ML) in hepatic disorders, the blue cluster concentrates on chronic liver diseases and cirrhosis research and the red cluster represents the core investigations on fatty liver and Non-Alcoholic Fatty Liver Disease (NAFLD). **(B)**: NAFLD Research Field Keyword Network Graph.This figure uses a color spectrum to represent temporal changes, displaying the co-occurrence network and connection strength of major keywords in NAFLD research, with “Machine Learning” and “NAFLD” as the core nodes. **(C)**: NAFLD Research Keyword Link Strength Heat Map.This figure shows the association strength between keywords using a red-blue spectrum, where red indicates high-strength associations and blue indicates low-strength associations, intuitively reflecting the closeness between research topics. **(D)**: Top 21 Keywords with the Highest Citation Frequency from 2010 to 2025. Arranged in chronological order, with citation burst periods marked by red lines.

Figure 8A demonstrates research topic groupings through multi-colored clustering, where node size represents keyword frequency, connection thickness indicates co-occurrence strength, and different colors represent 10 distinct research clusters. The central node “NAFLD” serves as the core research theme, forming close connections with technical keywords such as “Machine Learning,” “Artificial Intelligence,” and “Deep Learning.” With “Machine Learning” and “NAFLD” as the two largest central nodes, the green area representing traditional medical imaging technologies (ultrasound, MRI, elastography) is deeply integrating with the blue area's artificial intelligence technologies (machine learning, deep learning), forming new non-invasive diagnostic paradigms exemplified by radiomics, providing important alternatives to traditional liver biopsy diagnosis. The purple and red clusters highlight the importance of disease prediction models and epidemiological studies, particularly attention to special populations such as children.

Figures 8B,C displays temporal dynamics (2021.0–2024.0) and normalized citation strength (0.4–1.4) through gradient color spectra, presenting the temporal evolution of research hotspots and academic impact distribution. Particularly noteworthy in Figure 8B, keywords “Inflammation,” “CT,” and “Bioinformatics” appear in red, indicating high normalized citation impact. Figure 8C provides intuitive temporal evolution evidence. The timeline from left to right (2021–2024) shows distinct color gradation trends, with MASLD and MASH appearing in bright red, indicating the AI research community's rapid adaptation to new conceptual frameworks.

Keywords with high citation burst intensity are widely recognized as important indicators for identifying research frontier hotspots, effectively reflecting innovation dynamics and developmental trends in the field. The red bars in the figure represent citation burst periods for each keyword, with numerical values indicating burst strength, reflecting the academic evolution trajectory from basic metabolic mechanism research toward precision diagnosis and epidemiological investigation. Figure 8D displays the top 21 burst keywords from 2010 to 2025. “Hepatic steatosis” demonstrated the highest burst strength (6.94), followed by “classification” (3.64) and “fatty liver” (3.57). Additionally, “alanine aminotransferase” exhibited the longest burst duration, spanning 10 years. Notably, “immune infiltration” and “expression” emerged as the strongest recent research hotspots (2023–2025), with burst intensities of 2.46 and 3.29, respectively. This marks a transition from traditional imaging diagnosis and clinical manifestation studies toward deeper exploration of molecular biological mechanisms and immunological aspects. The appearance of “pathogenesis” (2.23) further confirms the research focus shift toward mechanistic analysis.

### 3.6 Citation analysis

**Figure 9:**
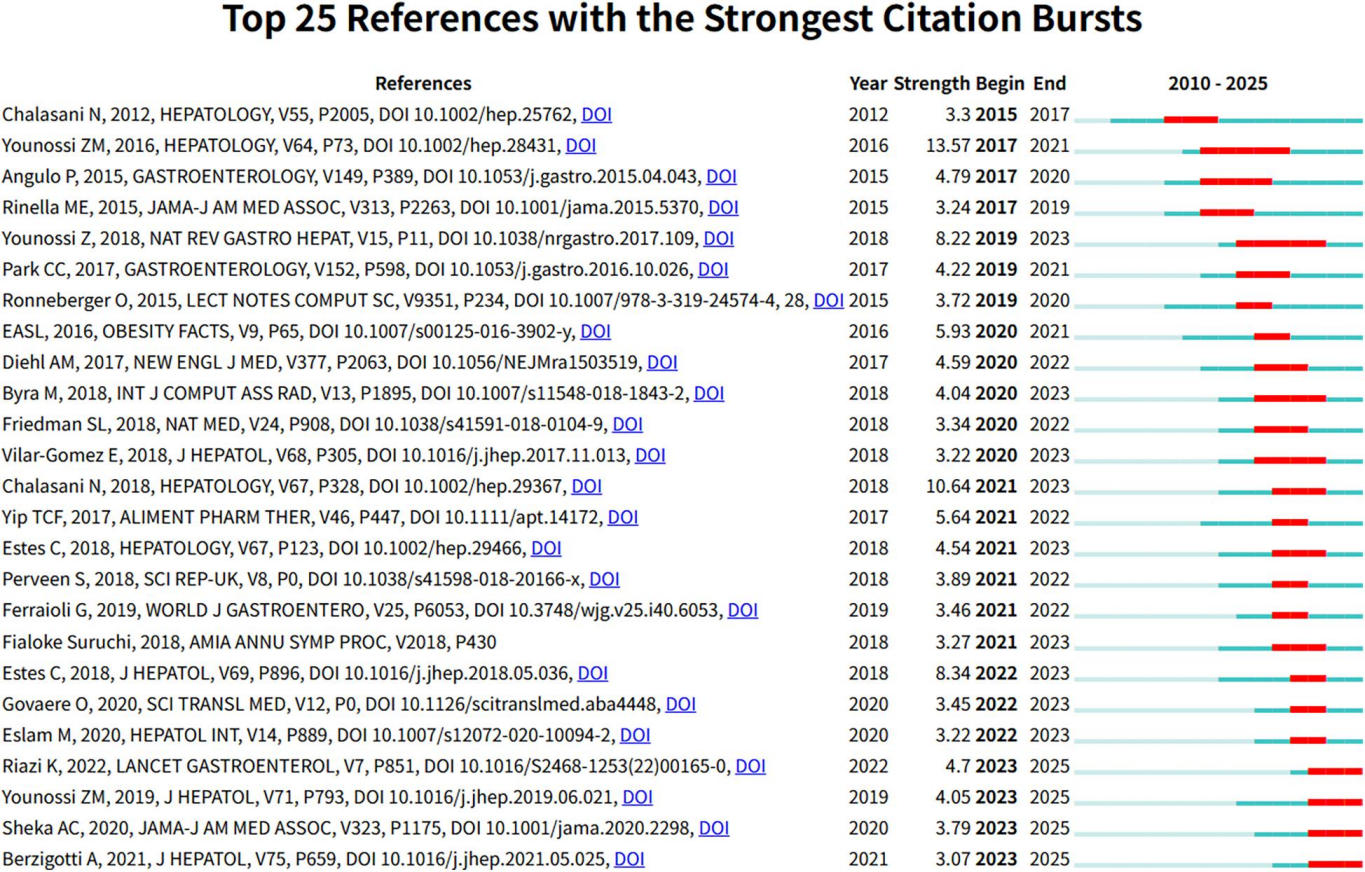
The 25 most significant citation burst papers in Non-alcoholic fatty liver disease research field. Figure shows the 25 papers with the strongest citation bursts in the NAFLD research field, including authors, publication year, journal, citation strength, and the start and end times of the citation burst.

**Figure 10:**
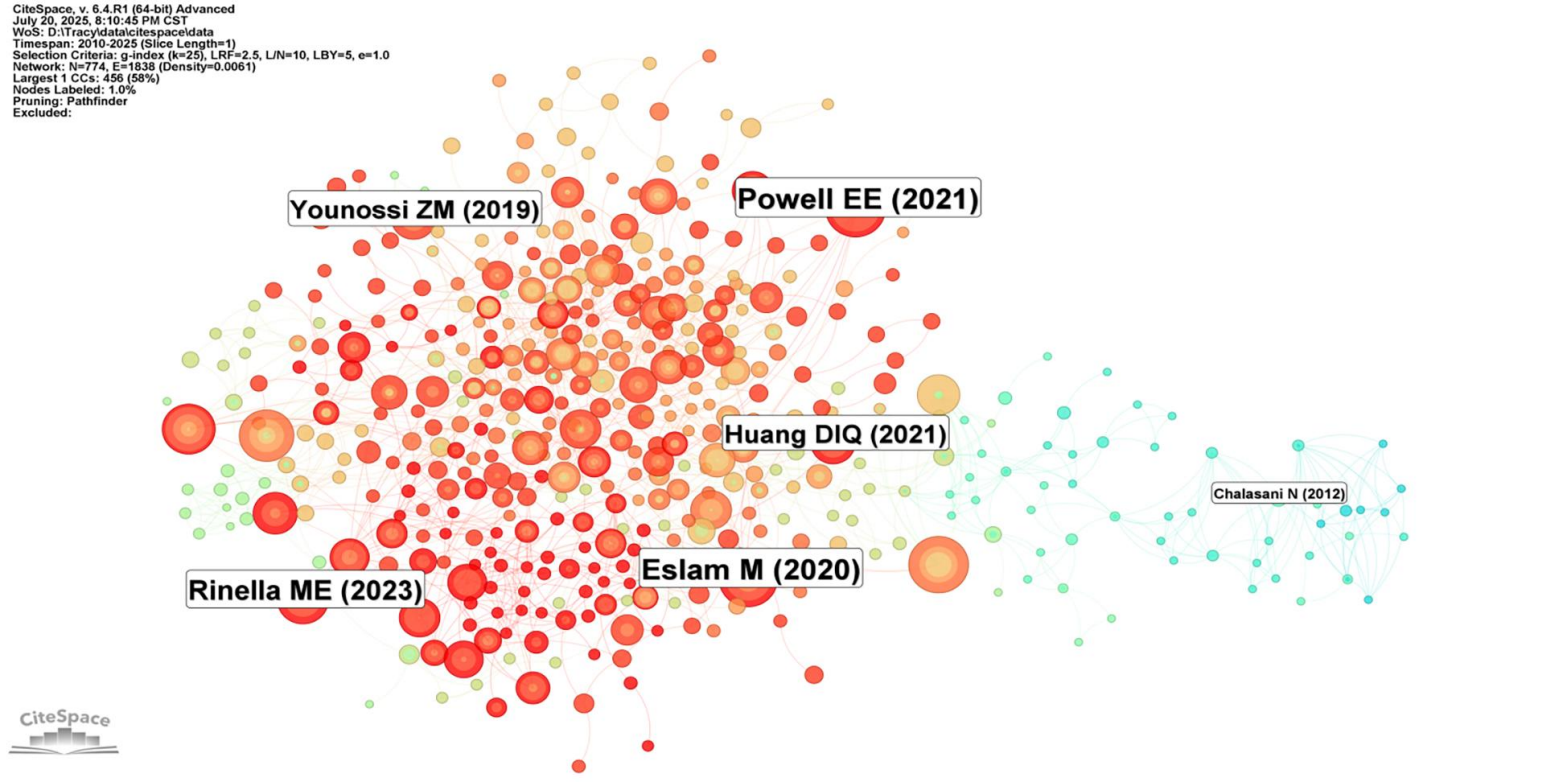
Citation network structure of Non-alcoholic fatty liver disease research. Figure presents a citation network visualization map of NAFLD research, where node size represents the influence of literature, and colors ranging from light green to deep red indicate publication times from early to recent periods. Although only some authors are labeled in the figure (such as Powell EE, Riazi K, etc.), the overall network structure reveals the clustering and knowledge flow directions in the research field.

Figure 9 presents the 25 references with the most significant citation bursts from 2010 to 2025. Among these, Younossi ZM's study published in Hepatology (2016) demonstrated the highest citation burst strength (26). Moreover, citation bursts of these high-impact publications were predominantly concentrated between 2020 and 2023. Figure 10 reveals academic associations among researchers through the CiteSpace citation network structure. Node size represents literature impact, while color variations indicate research hotspots migrating from early foundational studies (light green nodes) on the left to recent deep learning applications (dark red nodes) on the right, forming a distinct academic evolution pathway.

**Figure 11:**
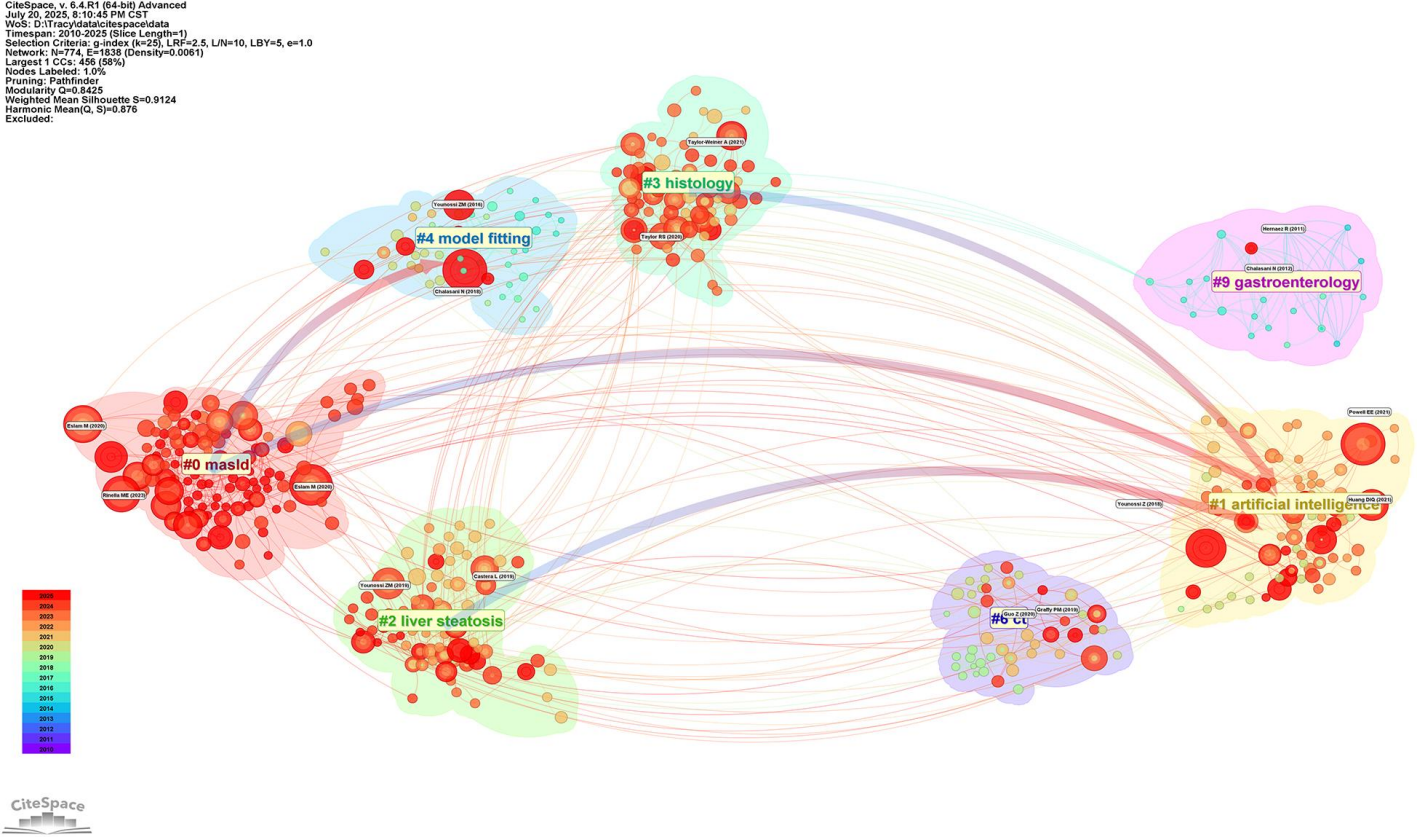
Thematic clustering network of Non-alcoholic fatty liver disease research. Figure displays the thematic clusters in NAFLD research and their interconnections. The figure identifies 12 major research directions, including masld(#0), artificial intelligence (#1), and hepatic steatosis (#2), among others. The connections between clusters reflect the cross-integration of research directions, especially the trend of integrating artificial intelligence technologies with traditional medical diagnostic methods.

Subsequently, this study conducted cluster analysis of cited journals (Figure 11), identifying 9 major research direction clusters including artificial intelligence (#1), hepatic steatosis (#2), histology (#3), model fitting (#4), among others. Inter-cluster connections demonstrate the cross-integration between artificial intelligence technologies and traditional medical diagnostic methods. Research outcomes published in these journals significantly advanced related research topics and achieved widespread citation.

**Figure 12:**
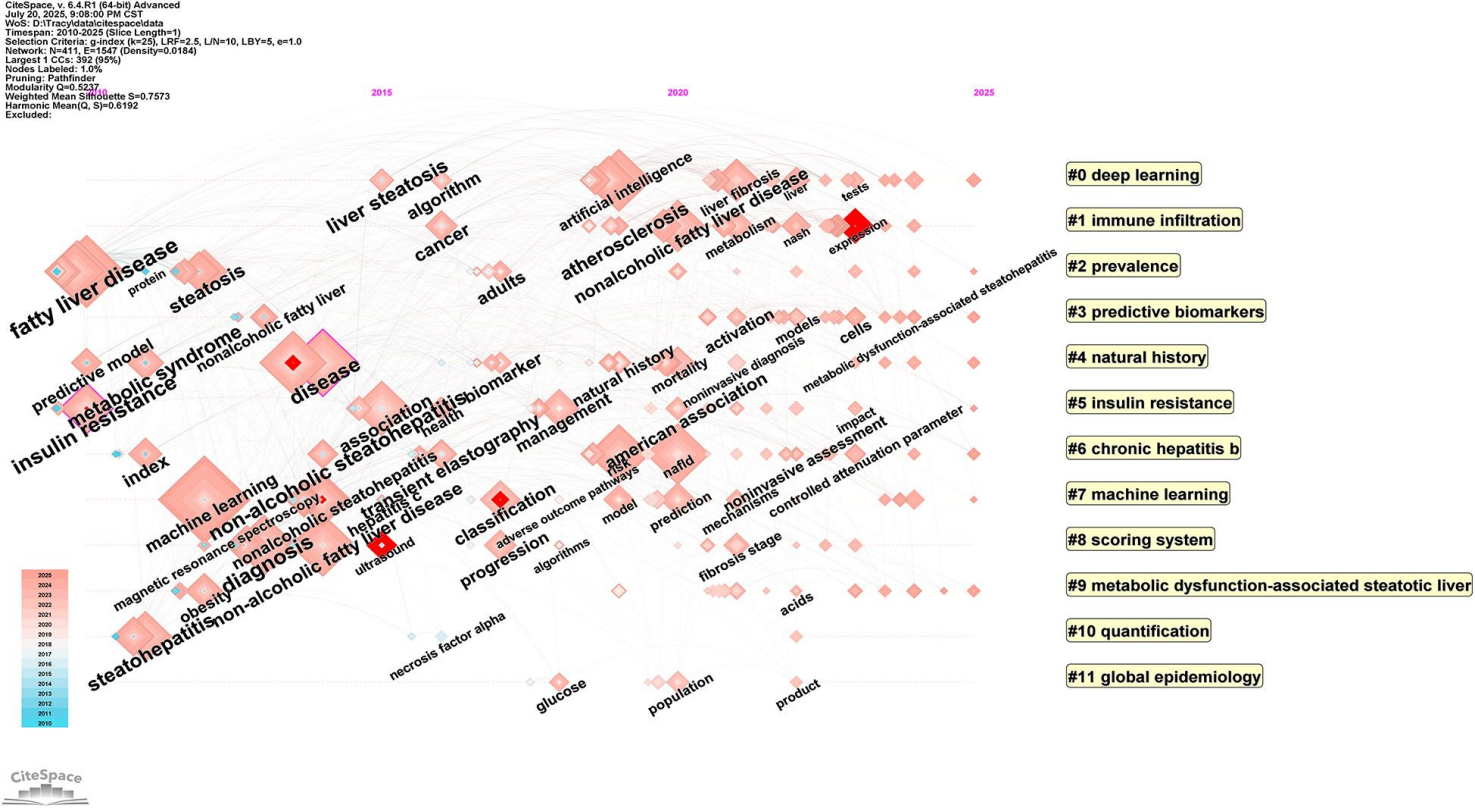
Timeline visualization Map of artificial intelligence applications in NAFLD. Through cluster analysis, cluster labels are generated, and references within clusters are arranged chronologically by publication year, with node size representing the total citation count of references. The horizontal axis represents time, with research progressing from early (left) to recent (right) periods.

The cited journal cluster timeline in Figure 12 displays dynamic evolution of research hotspots along the temporal axis (2010–2025). References within clusters are chronologically ordered by publication year, with node size representing total citation count. The horizontal axis represents time progression from early to recent research. Research themes evolved from 2010 to 2025, with early studies (2010) focusing on traditional pathophysiological mechanism exploration, mid-term development emphasizing clinical practice guidelines and diagnostic criteria, while recent studies (2020) clearly shifted toward deep learning and machine learning applications.

## 4 Discussion

### 4.1 Temporal evolution and research development trends in NAFLD-AI studies

This study systematically analyzed 655 publications retrieved from the period spanning 2010 to March 25, 2025, investigating the evolutionary trends of artificial intelligence applications in non-alcoholic fatty liver disease over the past fifteen years. Based on the time series regression analysis (Supplementary Table S1), this study identified a significant logarithmic-linear relationship between publication volume and the temporal variable (R^2^ = 0.943, F = 216.642, *p* *<* *0.001*). The regression coefficient *β* = 0.217 indicates an annual growth rate of 24.2% (e^0.217^ ≈ 1.242) in this field, demonstrating statistically significant growth patterns. The model's high explanatory power (adjusted R^2^ = 0.939) indicates that temporal factors account for 94.3% of publication volume variance, confirming sustained and stable exponential growth trends in this research domain.

Data analysis reveals that 2010–2018 represents the nascent phase of this field, characterized by relatively modest publication volumes with annual outputs not exceeding 10 articles. Subsequently, publication volumes demonstrated steady growth trajectories, with research scale rapidly expanding during 2019–2024, exhibiting exponential growth in publications (approximately 83.8% of total output). This pattern reflects the substantial attention and investment from the global scientific community toward AI-enabled NAFLD diagnosis and treatment.

Due to data cutoff limitations, the 2025 publication data (62 articles) represent only the first quarter of the year. Combined with temporal lag effects inherent in academic publishing, these incomplete data may underestimate actual research activity levels for that year. However, this limitation does not compromise the validity of the core conclusion identifying 2023–2024 as the breakthrough period for research in this domain.

Overall, the geographical distribution of research demonstrates a pronounced “Sino-American dual-core” configuration. The combined research contributions from these two nations account for 64.6% of the total output (Table 1). In contrast to the 64.6% research contribution from China and the United States, Europe, despite having the highest NAFLD prevalence rate globally (54.53%), contributes only 32.8% of research output, while representation from other high-prevalence Asian countries and regions remains notably limited. Based on collaboration network analysis, this imbalance reflects structural disparities in scientific resource allocation: AI medical research and development requires large-scale computational infrastructure, standardized datasets, and interdisciplinary talent teams—resources whose accessibility varies significantly across different regions. Specifically, resource constraints in underrepresented regions may impede the effective global dissemination of AI technologies. Consequently, the current research concentration phenomenon not only reflects technological development inequalities but may also exacerbate disparities in NAFLD precision diagnostic and therapeutic capabilities across different regions. To achieve equitable global development of AI-assisted NAFLD management, future research should prioritize establishing more inclusive international collaboration mechanisms, particularly strengthening partnerships with regions bearing high disease burdens but possessing relatively limited research resources, ensuring that AI technological innovations benefit global patient populations.

Beyond these findings, this study reveals that the United States' academic impact superiority in NAFLD artificial intelligence research stems from three core mechanisms: First, systematic differences in research quality—American institutions such as the University of Wisconsin-Madison, despite limited publication volume (18 articles), established standardized validation systems in CT imaging analysis and Rohit Loomba's interdisciplinary research platform, ensuring methodological rigor and clinical translational value. Second, the knowledge amplification effect of diversified international collaboration networks—deep partnerships between the United States and major European research nations enhanced international visibility and citation dissemination of research outcomes. Third, the citation accumulation advantage of early technological positioning—American research teams' early exploration in the AI-NAFLD field established important methodological foundations and validation standards, with these pioneering works becoming essential citation sources for subsequent research, thereby forming sustained influence accumulation within citation networks. In contrast, China, leveraging its substantial research scale and rapid development of emerging institutions, demonstrates unique advantages in large-sample data acquisition and technological iteration, indicating the formation of future multipolar collaboration patterns.

Following analysis of overall research trends, we subsequently examined academic publication patterns and journal distribution characteristics in this field. Journal analysis can reflect the interdisciplinary nature of a field and changes in publication patterns, guiding clinical practitioners and researchers in journal selection and identifying emerging research areas. Scientific Reports (43 articles, 6.6%), as the journal with the highest publication volume, has become an important publication platform for this emerging interdisciplinary field due to its open-access model and interdisciplinary characteristics. Core journals including Scientific Reports, Frontiers in Endocrinology, and Diagnostics have published substantial AI-NAFLD related research in recent years (2022–2024). These journals typically offer faster publication speeds and broader accessibility.

### 4.2 Evolutionary trajectory and core application patterns of AI technologies in the NAFLD domain

Based on comprehensive evidence from keyword burst analysis, citation network evolution, and author collaboration patterns, AI applications in the NAFLD field have undergone a distinct three-stage technological evolution. Each phase represents different algorithmic approaches, research priorities, and technical challenges that reflect the maturation of AI methodologies in clinical medicine.

The Traditional Machine Learning Era (2010–2018) was characterized by the systematic processing of structured clinical data. Keyword burst analysis demonstrates that “alanine aminotransferase” maintained the longest burst duration (10 years), while traditional biomarkers such as “insulin resistance” dominated the research landscape. This pattern indicates that early AI applications primarily focused on data mining of conventional laboratory parameters and the development of risk stratification models. The predominant computational approaches during this period included Support Vector Machines (SVM), Decision Trees, and Logistic Regression (27, 28). These traditional machine learning methods offered distinct advantages in terms of model interpretability and robust performance with limited sample sizes—critical considerations for early clinical AI applications.Citation analysis provides additional insights into the academic foundation established during this era. Among the 25 publications demonstrating the most significant citation bursts, the work by Younossi ZM (2016) achieved the highest citation intensity of 13.57. This landmark study conducted a comprehensive global meta-analysis that systematically characterized the worldwide epidemiological patterns of NAFLD, thereby establishing essential epidemiological foundations for subsequent AI-driven risk prediction and population screening applications.

The Deep Learning Breakthrough Era (2019–2022) represented a paradigm shift from clinical parameter analysis toward sophisticated medical imaging processing. Citation burst analysis reveals concentrated activity in keywords including “U-Net deep learning architecture,” “convolutional neural networks,” “artificial intelligence,” and “segmentation,” signaling the emergence of breakthrough applications in medical imaging analysis. The red research cluster centered on Pickhardt (16 publications, 843 total citations) has predominantly focused on artificial intelligence applications in computed tomography. Their developed AI-automated CT analysis tools have demonstrated exceptional performance in detecting moderate-to-severe hepatic steatosis. When employing a diagnostic threshold of liver contrast-enhanced attenuation values below 90 HU, the system achieved a sensitivity of 90.5% and specificity of 78.4%. With a more stringent threshold of <80 HU, sensitivity was 77.8% while specificity improved to 93.2%. The diagnostic performance yielded an AUC of 0.938, providing a reliable automated solution for opportunistic fatty liver screening in routine CT examinations (29).

The Multimodal Integration Era (2023–2025) has been characterized by AI applications extending from imaging diagnostics into molecular biological mechanisms. Keyword burst analysis demonstrates (as shown in Figure 8D) that “immune infiltration” (burst strength 3.29) and “gene expression” (burst strength 2.46) have emerged as the most prominent recent research hotspots. This transition marks an evolution from traditional imaging diagnosis and clinical manifestation studies toward deeper exploration of molecular biological mechanisms and immunological aspects.AI technologies have demonstrated unique value in immune infiltration quantification. Machine learning algorithms can automatically quantify infiltration patterns of key immune cells including macrophages and regulatory T cells, revealing dynamic associations with metabolic inflammation (30). Deep learning approaches enable precise identification of inflammatory cell spatial distribution through histological image analysis, providing quantitative foundations for understanding immune crosstalk between metabolic organs (31). This AI-driven quantification of immune infiltration represents a critical technology for elucidating adipokine-mediated inter-organ immune dialogue mechanisms, offering essential tools for developing precision intervention strategies targeting metabolic inflammation.

In thematic cluster analysis (Figure 8A), “NAFLD” and “Machine Learning” constitute the core conceptual framework as the most frequently occurring keywords. The biological foundation underlying these technological applications is profound: research demonstrates that adipose tissue coordinates “meta-inflammation” responses through adipokine release. This inflammatory state represents a common characteristic of metabolic diseases and directly promotes NAFLD progression (32). AI technologies achieve precision diagnosis and prediction by quantifying these complex biological processes.

The temporal distribution of citation bursts further elucidates the paradigmatic shifts in research priorities within this domain: progressing from early emphasis on clinical parameters and pathological characteristics, through the intermediate development of risk prediction models, to the contemporary widespread application of deep learning technologies. Moreover, co-citation network analysis (Figures 10–12) corroborates this evolutionary trajectory, demonstrating the migration of research foci from foundational studies toward advanced deep learning applications, thereby establishing a coherent academic progression pathway.

The nine principal research directions identified through cluster analysis constitute a comprehensive knowledge landscape of this field. Temporal axis analysis confirms the systematic evolution of research themes from epidemiological and foundational concepts, through clinical practice guidelines and diagnostic criteria, ultimately transitioning toward deep learning and precision medicine applications. Of particular significance is the prominent emergence of metabolic dysfunction-associated fatty liver disease (#9) in recent years, which correlates closely with the MAFLD nomenclature transition. The knowledge mapping demonstrates rapid growth in MAFLD-related research post-2020, alongside close associations with clusters encompassing insulin resistance and predictive biomarkers. This pattern substantively reflects the profound impact of this conceptual transformation on research trajectories. The nomenclature revision has facilitated a research paradigm shift from merely excluding alcoholic factors toward actively identifying metabolic risk factors, thereby advancing the development of artificial intelligence-based precision stratification diagnostics and individualized therapeutic strategies.This conceptual evolution has provided essential theoretical foundations and practical guidance for research paradigm transformation in this field. The transition emphasizes the movement from exclusion-based diagnostic approaches toward comprehensive metabolic risk assessment, aligning with contemporary precision medicine principles and establishing a robust framework for future AI-driven clinical applications in NAFLD management.

Based on the aforementioned technological evolution analysis and temporal distribution characteristics of citation bursts, this study further reveals that artificial intelligence applications in NAFLD research are primarily concentrated in three domains: non-invasive diagnostic technology development, predictive model construction, and biomarker identification. Machine learning and deep learning methodologies have become core methodological tools in NAFLD research, introducing new technological paradigms to traditional clinical investigations. The application of deep learning algorithms has enabled highly automated diagnostic processes for NAFLD. This technological innovation has substantially enhanced the capacity for early detection of clinically significant NAFLD, providing possibilities for timely intervention.

In terms of specific algorithmic applications, deep learning algorithms have achieved high automation in NAFLD diagnostic processes. Taking NASH-Scope (also known as Fibro-Scope) as an example, this artificial neural network diagnostic system demonstrated excellent performance in validation studies: sensitivity of 97.2% and specificity of 97.8% (AUC = 0.950) for distinguishing NAFLD from healthy controls, and sensitivity of 90.7% with specificity of 93.3% for identifying NASH with fibrosis. In fibrosis staging, the system achieved sensitivity of 99.5% and specificity of 90.9% for distinguishing F0 from F1-4 stages, providing a reliable clinical tool for non-invasive NAFLD screening and fibrosis assessment (33, 34).

Beyond diagnostic applications, artificial intelligence technologies have demonstrated exceptional potential in NAFLD risk prediction. The predictive model developed by Charu et al. (35) based on the “super learner” algorithm has shown outstanding performance in detecting fibrotic NASH. This type of artificial intelligence model integrates the advantages of multiple machine learning algorithms to form “best-in-class” predictors, providing novel benchmark tools for clinical risk assessment.Artificial intelligence technologies also play a crucial role in hepatic steatosis evaluation. Neural network-based ultrasound assessment methods analyze high-level features extracted from hepatic B-mode ultrasound image sequences through deep convolutional neural networks (36), enabling automated assessment of hepatic fat content and effectively reducing clinical workload.

In biomarker identification, artificial intelligence similarly demonstrates broad prospects. Through the application of machine learning algorithms to construct MAFLD models and screen disease-characteristic genes, potential therapeutic targets can be identified, achieving goals of prediction, prevention, and personalized treatment (37). Currently developed quantitative imaging biomarkers include liver elastography for fibrosis staging and magnetic resonance proton density fat fraction for hepatic steatosis assessment (38). These technologies provide novel pathways for predicting the risk of early chronic liver disease progression to cirrhosis-related complications, facilitating the realization of precision medicine. Furthermore, machine learning algorithms have been applied to quantitative assessment of NAFLD immune cell infiltration patterns (39), providing new perspectives for disease mechanism research.

### 4.3 Summary and future perspectives of AI applications in NAFLD research

Currently, liver tissue biopsy remains the gold standard for NAFLD diagnosis, staging, and prognostic assessment (40, 41). However, this approach presents significant limitations: it is not only invasive but may also induce rare yet life-threatening complications, while facing challenges including sampling variability and high costs (42). These constraints limit the widespread application of liver biopsy in large-scale population screening and long-term follow-up studies.

The advancement of artificial intelligence technologies offers a breakthrough solution to this clinical dilemma.Although non-invasive methods such as FibroScan and ELF serum testing have been implemented in clinical practice, each approach possesses specific limitations. Elastography techniques demonstrate reduced success rates in obese patients and are susceptible to acute inflammation effects, while serum biomarker detection, despite higher standardization levels, exhibits limited sensitivity in early fibrosis detection (42). Artificial intelligence technologies demonstrate the potential to integrate clinical parameters, laboratory indices, and imaging features, promising to further enhance diagnostic performance beyond existing non-invasive methodologies (38). Non-invasive diagnosis and precision staging represent promising application directions for artificial intelligence technologies in the NAFLD domain (36). Accurate assessment of hepatic fibrosis is not only crucial for predicting disease outcomes in clinical practice but also holds irreplaceable value in evaluating therapeutic responses within clinical trials (43).

The MAESTRO-NASH large-scale randomized controlled trial has further validated the clinical utility of non-invasive diagnostic technologies. This landmark study demonstrated that multiple non-invasive assessment methods, including blood biomarkers and imaging examinations, possess sufficient sensitivity and specificity to accurately evaluate NASH disease activity and fibrosis severity (44). This milestone clinical evidence provides essential validation benchmarks for further optimization and clinical translation of AI technologies, particularly in constructing more precise predictive models and risk stratification systems.These developments underscore the transformative potential of artificial intelligence in revolutionizing NAFLD clinical management through enhanced diagnostic accuracy, reduced invasiveness, and improved patient accessibility to comprehensive hepatological assessment.

Despite the tremendous potential demonstrated by artificial intelligence in NAFLD research, bibliometric analysis from this study reveals that current research confronts three core challenges requiring urgent resolution.First, the absence of standardized datasets constitutes a fundamental constraint limiting large-scale technological applications (45). Most contemporary studies employ datasets exhibiting significant heterogeneity in image acquisition protocols, pathological diagnostic criteria, and patient population characteristics, thereby compromising model reproducibility and generalizability (10). This phenomenon is exemplified by the highest publication volumes observed in open-access journals such as Scientific Reports, where research quality demonstrates considerable variability and lacks unified data standards and validation benchmarks. The establishment of multi-center, large-sample, standardized NAFLD imaging and pathological databases represents a foundational prerequisite for achieving AI technological breakthroughs. Second, limitations in external validation significantly compromise the credibility of research findings. This investigation reveals that most high-impact publications undergo validation exclusively within single institutions or specific populations, lacking cross-racial and cross-regional external cohort validation. This constraint substantially limits the applicability of AI models across diverse clinical environments, thereby hindering their broader implementation.

Additionally, citation burst analysis from this study illuminated the phenomenon of “technological translation delay.” For instance, the U-Net deep learning architecture proposed by Ronneberger O in 2015 did not trigger citation bursts in NAFLD research until 2019–2020 (burst intensity 3.72) (46), indicating an approximate 4–5 year translation cycle from artificial intelligence theoretical development to practical medical applications. This temporal lag stems not only from technological maturity considerations but also reflects systemic barriers including healthcare workflow adaptation requirements, clinical physician acceptance cultivation, and regulatory approval complexities. These findings underscore the necessity for systematic approaches to address infrastructure, validation, and implementation challenges to fully realize the transformative potential of artificial intelligence in NAFLD clinical practice.

To accelerate the clinical implementation of AI technologies in NAFLD practice, comprehensive advancement is required across four strategic dimensions: data resource integration and standardization, institutionalization of multidisciplinary collaboration mechanisms, adaptive reform of regulatory and evaluation frameworks, and systematic development of international cooperation networks.

Based on successful experiences from high-impact research institutions, we propose four specific improvement strategies. First, data resource integration and standardization represents the foundational prerequisite for eliminating translational bottlenecks. Examining successful models from high-impact institutions, the “gold standard” validation framework established by the University of Wisconsin-Madison in CT imaging analysis (with research primarily focused on CT-based hepatic fat quantification technology development, including fully automated deep learning segmentation tools and opportunistic screening methods) and the interdisciplinary standardized research platform developed by Rohit Loomba's team at the University of California San Diego both exemplify the critical importance of data standardization for rapid technology validation.

Second, institutionalized innovation in multidisciplinary collaboration mechanisms holds decisive significance for overcoming traditional developmental bottlenecks. High-productivity author network analysis from this study reveals that successful research teams universally establish deep integration models between clinical medicine and computational sciences. This collaborative paradigm ensures close alignment between AI algorithm development and clinical requirements, thereby significantly reducing the temporal span from proof-of-concept to clinical application.

Third, adaptive reform of regulatory and evaluation frameworks should be optimized according to the technical characteristics of AI medical applications. For medium-to-low risk clinical applications such as NAFLD diagnosis, we recommend implementing risk-stratified differential management strategies through the establishment of experimental clinical application evaluation mechanisms that permit limited-scope clinical validation while ensuring patient safety.

Fourth, systematic development of international cooperation networks will further optimize translational efficiency through knowledge sharing and resource integration. The close collaborative relationships between the United States and major research nations including China and the United Kingdom provide foundations for rapid technology dissemination. Building upon this foundation, more institutionalized collaborative mechanisms should be established, including unified algorithm validation standards, data format specifications, and clinical evaluation guidelines, with the objective of reducing technological translation cycles to more efficient timeframes.

In summary, based on the analytical findings of this study, future research should strengthen collaborative exchanges with low- and middle-income countries where artificial intelligence technologies and resources remain limited, thereby promoting globally balanced development in this field. Concurrently, it is imperative to further dismantle disciplinary barriers and foster deep integration among imaging analysis, genomics, and clinical diagnostics, constructing a more innovative research ecosystem. Reducing the translational cycle from theoretical AI development to practical medical applications while accelerating technology implementation remains critically important.

Furthermore, enhanced application of AI technologies in NAFLD molecular pathology and immunological mechanism research should be prioritized to deepen understanding of disease pathogenesis. Achieving optimal balance between the practical utility of clinical prediction tools and their academic impact will facilitate synergistic development between fundamental research and clinical applications. These collective efforts will contribute to fully realizing the potential of artificial intelligence in NAFLD research and clinical practice, ultimately improving diagnostic accuracy and therapeutic outcomes for patients.

The advancement of AI-enabled NAFLD management represents a paradigmatic shift toward precision medicine, necessitating systematic coordination across technological, institutional, and regulatory domains. Through strategic implementation of the proposed multi-dimensional framework, the field can accelerate the translation of artificial intelligence innovations into clinically meaningful improvements in patient care,establishing a sustainable foundation for continued advancement in hepatological practice and research.

## 5 Conclusion

This study reveals three core findings regarding artificial intelligence applications in NAFLD research through comprehensive bibliometric analysis. First, the research landscape demonstrates a Sino-American dual-core driven multidisciplinary collaborative network, characterized by exponential growth patterns across three distinct phases: the nascent period (2010–2018), rapid growth period (2019–2022), and explosive development period (2023–2024). This evolution has established four major technological clusters: non-invasive assessment of hepatic fibrosis, imaging-based diagnosis, disease progression predictive modeling, and biomarker screening. Second, the research paradigm has undergone dual transformations—scientific inquiry has shifted from clinical phenotypic analysis toward molecular mechanism exploration, while technological approaches have evolved from single algorithms toward multimodal deep learning systems. Third, the explosive growth of emerging themes including “immune infiltration” and “gene expression” (2023–2025) signifies the field's entry into a new era of precision medicine.

Current AI technologies have achieved breakthroughs across three major application scenarios: deep learning-based imaging analysis has significantly enhanced non-invasive diagnostic accuracy for fatty liver disease, multidimensional machine learning models have enabled precise risk stratification, and bioinformatics algorithms have accelerated novel biomarker discovery. Despite confronting challenges related to data heterogeneity and clinical translation bottlenecks, AI technologies are reconstructing NAFLD diagnostic and therapeutic frameworks through innovations such as promoting non-invasive testing as liver biopsy alternatives and implementing quantitative predictive models to support clinical decision-making.

Future breakthrough directions should focus on: constructing standardized multimodal databases, developing interpretable AI systems, and establishing clinical validation frameworks. The longitudinal trend analysis and knowledge mapping constructed in this study provide strategic navigation for the deep integration of artificial intelligence and hepatology, substantially advancing precision hepatology development and contributing to addressing NAFLD as a global public health challenge.These findings underscore the transformative potential of artificial intelligence in revolutionizing NAFLD research methodologies and clinical practice, establishing a robust foundation for continued advancement in precision medicine approaches to metabolic liver disease management.

## 6 Limitations and Future Perspectives

This study possesses certain inherent limitations that warrant acknowledgment. First, potential selection bias may exist in the results due to the exclusive utilization of the WoSCC database and the exclusion of non-English publications, potentially overlooking significant research published in journals not indexed by Web of Science. Second, temporal constraints arise from the incomplete nature of 2025 data, which, given the rapid evolution of AI technologies, may compromise accurate assessment of the most recent trends.

From a methodological perspective, while bibliometric approaches excel in revealing macroscopic patterns and developmental trajectories, they possess inherent limitations in reflecting specific research quality and innovation. It is particularly important to note that the current study primarily employed descriptive statistics and visualization techniques. Although this methodological choice aligns with established traditions in exploratory bibliometrics, it indeed constrains the capacity for statistical validation of certain specific assertions, such as technology adoption cycles and the significance of international collaboration patterns.

Despite these limitations, we validated the representativeness of our findings through analysis of the disciplinary distribution of indexed journals. The included studies encompassed core journals across relevant fields, including premier hepatology journals (Hepatology, IF = 13.0) and AI medical journals (Artificial Intelligence in Medicine, IF = 6.1), with a broad distribution of impact factors, indicating robust disciplinary representativeness of our sample.

Future research should explore analytical frameworks that integrate descriptive bibliometrics with inferential statistical methods, while considering the incorporation of multiple data sources combined with qualitative assessment approaches. Such methodological advancement would provide more rigorous quantitative evidence and comprehensive understanding for this rapidly evolving field, thereby enhancing the precision and depth of insights into AI applications in NAFLD research and clinical practice.

## Data Availability

The original contributions presented in the study are included in the article/Supplementary Material, further inquiries can be directed to the corresponding author.

## Supplementary material

Supplementary Table 1 **Time Series Regression Analysis of Publication Growth Trends in Artificial Intelligence Applications for Non–Alcoholic Fatty Liver Disease Research (2010–2024).** Analysis includes complete annual data from 2010–2024 (*n *= 15 years). Data from 2025 were excluded due to incomplete year coverage (only partial data available through March 25, 2025). Dependent variable: ln_articles (natural logarithm of annual publication count). Independent variable: Time coding (2010 = 1, 2011 = 2, ... , 2024 = 15). Model interpretation: The regression coefficient *β* = 0.217 indicates an annual growth rate of 24.2% [e^0.217 ≈ 1.242]. Model performance: The model explains 94.3% of the variance in publication volume over the 15–year study period. Statistical assumptions: All regression assumptions were verified including normality of residuals, homoscedasticity, and independence of observations. Significance levels: *p* < 0.05, **p* < 0.01, ***p* < 0.001. Analysis software: IBM SPSS Statistics 26.0 using Enter method with *α* = 0.05.

Supplementary Table 2 **Identification of Core Authors Based on Co-citation Analysis.** This table presents the top 10 core authors identified through co-citation analysis in the field of artificial intelligence applications in non-alcoholic fatty liver disease research. Co-citations represent the frequency with which each author's work was cited alongside other authors' publications, indicating collaborative relationships and shared research themes. Total link strength measures the overall connectivity of each author within the citation network, with higher values indicating greater influence and centrality in the research community.

Supplementary Figure 1 **Time Evolution of Institutional Collaboration Networks in Artificial Intelligence Research on Non-alcoholic Fatty Liver Disease from 2021-2024.** Nodes represent research institutions, connecting lines represent collaborative relationships, and the color gradient from blue to red represents the temporal change from 2021 to 2024. This figure displays the institutional collaboration network for research related to non-alcoholic fatty liver disease (NAFLD) and artificial intelligence, presented in the form of a heat map. In the figure, each node represents a different research institution, while the lines between nodes indicate collaborative relationships between institutions; thicker lines represent stronger collaborative intensity. The color gradient from blue to red corresponds to the time span from 2021 to 2024, with red nodes indicating more active institutions in recent years (2023-2024), while blue nodes represent major participants in earlier years (2021-2022).

Supplementary Figure 2 **Visualization of institutional collaboration network in the field of non-alcoholic fatty liver disease and artificial intelligence research (bright background).** This network diagram shows collaborative relationships between research institutions, where nodes represent different institutions and connecting lines indicate collaborative relationships. Researchers such as Younossi ZM, Chalasani N, and others located in the central area occupy core positions in the network, indicating their significant influence in this field. Different colored clusters represent different research communities or directions.

Supplementary Figure 3 **Visualization of institutional collaboration network in the field of non-alcoholic fatty liver disease and artificial intelligence research (dark background).** The diagram clearly shows the collaborative relationships between the research team centered around Younossi ZM and other research groups such as Chalasani N, Pickhardt PJ, etc. Different colors represent the distribution of different research communities.

Supplementary Figure 4 **Research Topic Distribution Map in the Field of Non-alcoholic Fatty Liver Disease and Artificial Intelligence Research.** This network diagram illustrates the thematic distribution and interconnections within the research domains of non-alcoholic fatty liver disease and artificial intelligence. The nodes represent distinct research directions or keywords, while the connecting lines indicate the relationships among these topics. Multiple research clusters are distributed around the core area of “Scientific Reports,” encompassing hepatology, diagnostics, imaging, artificial intelligence applications, and related clinical fields. The color gradient from blue to red reflects the temporal evolution or research intensity changes of topics spanning from 2021 to 2024.
