# Supplementary material for: The evolution of artificial intelligence technology in non-alcoholic fatty liver disease
Source: Front Radiol. 2025 Sep 16;5:1634165. doi: 10.3389/fradi.2025.1634165 (PMC12480972; doi:10.3389/fradi.2025.1634165)
Supplement: Supplementary Table 1 — Time Series Regression Analysis of Publication Growth Trends in Artificial Intelligence Applications for Non–Alcoholic Fatty Liver Disease Research (2010–2024). Analysis includes complete annual data from 2010–2024 (n = 15 years). Data from 2025 were excluded due to incomplete year coverage (only partial data available through March 25, 2025). Dependent variable: ln_articles (natural logarithm of annual publication count). Independent variable: Time coding (2010 = 1, 2011 = 2, ... , 2024 = 15). Model interpretation: The regression coefficient β = 0.217 indicates an annual growth rate of 24.2% [e^0.217 ≈ 1.242]. Model performance: The model explains 94.3% of the variance in publication volume over the 15–year study period. Statistical assumptions: All regression assumptions were verified including normality of residuals, homoscedasticity, and independence of observations. Significance levels: p < 0.05, *p < 0.01, **p < 0.001. Analysis software: IBM SPSS Statistics 26.0 using Enter method with α = 0.05. [file Table1.docx]

**Supplementary Table 1.Time Series Regression Analysis of Publication Growth Trends in Artificial Intelligence Applications for Non-Alcoholic Fatty Liver Disease Research (2010-2024).**

| **Analysis Component** | **Parameter** | **Value** | **95% CI** | **Statistical Significance** |
| --- | --- | --- | --- | --- |
| **Model Summary** |  |  |  |  |
|  | Correlation coefficient (R) | 0.971 | - | - |
|  | Coefficient of determination (R²) | 0.943 | - | - |
|  | Adjusted R² | 0.939 | - | - |
|  | Standard error of estimate | 0.400 | - | - |
| **Analysis of Variance** |  |  |  |  |
|  | F-statistic | 216.642 | - | p < 0.001*** |
|  | Regression sum of squares | 34.703 | - | - |
|  | Residual sum of squares | 2.082 | - | - |
|  | Total sum of squares | 36.786 | - | - |
|  | Degrees of freedom (regression) | 1 | - | - |
|  | Degrees of freedom (residual) | 13 | - | - |
| **Regression Coefficients** |  |  |  |  |
|  | Intercept (constant) | -0.262 | [-0.378, -0.146] | p = 0.251 |
|  | Time coding coefficient (β) | 0.217 | [0.188, 0.246] | p < 0.001*** |
|  | Standard error (β) | 0.024 | - | - |
|  | t-statistic | 14.719 | - | - |
|  | Standardized coefficient (Beta) | 0.971 | - | - |
| **Model Diagnostics** |  |  |  |  |
|  | Variance inflation factor (VIF) | 1.000 | - | No multicollinearity |
|  | Tolerance | 1.000 | - | - |
|  | Condition index | 3.956 | - | Acceptable |
|  | Durbin-Watson statistic | - | - | Assumed independent |

**Study period: Analysis includes complete annual data from 2010-2024 (n=15 years). Data from 2025 were excluded due to incomplete year coverage (only partial data available through March 25, 2025)Dependent variable: ln_articles (natural logarithm of annual publication count)Independent variable: Time coding (2010=1, 2011=2, ..., 2024=15)Model interpretation: The regression coefficient β = 0.217 indicates an annual growth rate of 24.2% [e^0.217 ≈ 1.242]Model performance: The model explains 94.3% of the variance in publication volume over the 15-year study periodStatistical assumptions: All regression assumptions were verified including normality of residuals, homoscedasticity, and independence of observationsSignificance levels: p < 0.05, *p < 0.01, **p < 0.001Analysis software: IBM SPSS Statistics 26.0 using Enter method with α = 0.05.**
