# Supplementary material for: The evolution of artificial intelligence technology in non-alcoholic fatty liver disease
Source: Front Radiol. 2025 Sep 16;5:1634165. doi: 10.3389/fradi.2025.1634165 (PMC12480972; doi:10.3389/fradi.2025.1634165)
Supplement: Supplementary Table 2 — Identification of Core Authors Based on Co-citation Analysis. This table presents the top 10 core authors identified through co-citation analysis in the field of artificial intelligence applications in non-alcoholic fatty liver disease research. Co-citations represent the frequency with which each author's work was cited alongside other authors' publications, indicating collaborative relationships and shared research themes. Total link strength measures the overall connectivity of each author within the citation network, with higher values indicating greater influence and centrality in the research community. [file Table2.docx]

**Supplementary Table 2 .Identification of Core Authors Based on Co-citation Analysis.**

| **Author** | **co-citations** | **Total link strength** |
| --- | --- | --- |
| Younossi, Zm | 456 | 4438 |
| Pickhardt, Pj | 180 | 1795 |
| Kleiner, De | 163 | 2003 |
| Rinella, Me | 137 | 1537 |
| Eslam, M | 135 | 1124 |
| Chalasani, N | 131 | 1226 |
| Angulo, P | 130 | 1664 |
| Loomba, R | 130 | 1724 |
| Brunt, Em | 128 | 1685 |
| Bedossa, P | 105 | 1372 |

This table presents the top 10 core authors identified through co-citation analysis in the field of artificial intelligence applications in non-alcoholic fatty liver disease research. Co-citations represent the frequency with which each author's work was cited alongside other authors' publications, indicating collaborative relationships and shared research themes. Total link strength measures the overall connectivity of each author within the citation network, with higher values indicating greater influence and centrality in the research community.
