# Supplementary figures and images for: The evolution of artificial intelligence technology in non-alcoholic fatty liver disease
Source: Front Radiol. 2025 Sep 16;5:1634165. doi: 10.3389/fradi.2025.1634165 (PMC12480972; doi:10.3389/fradi.2025.1634165)

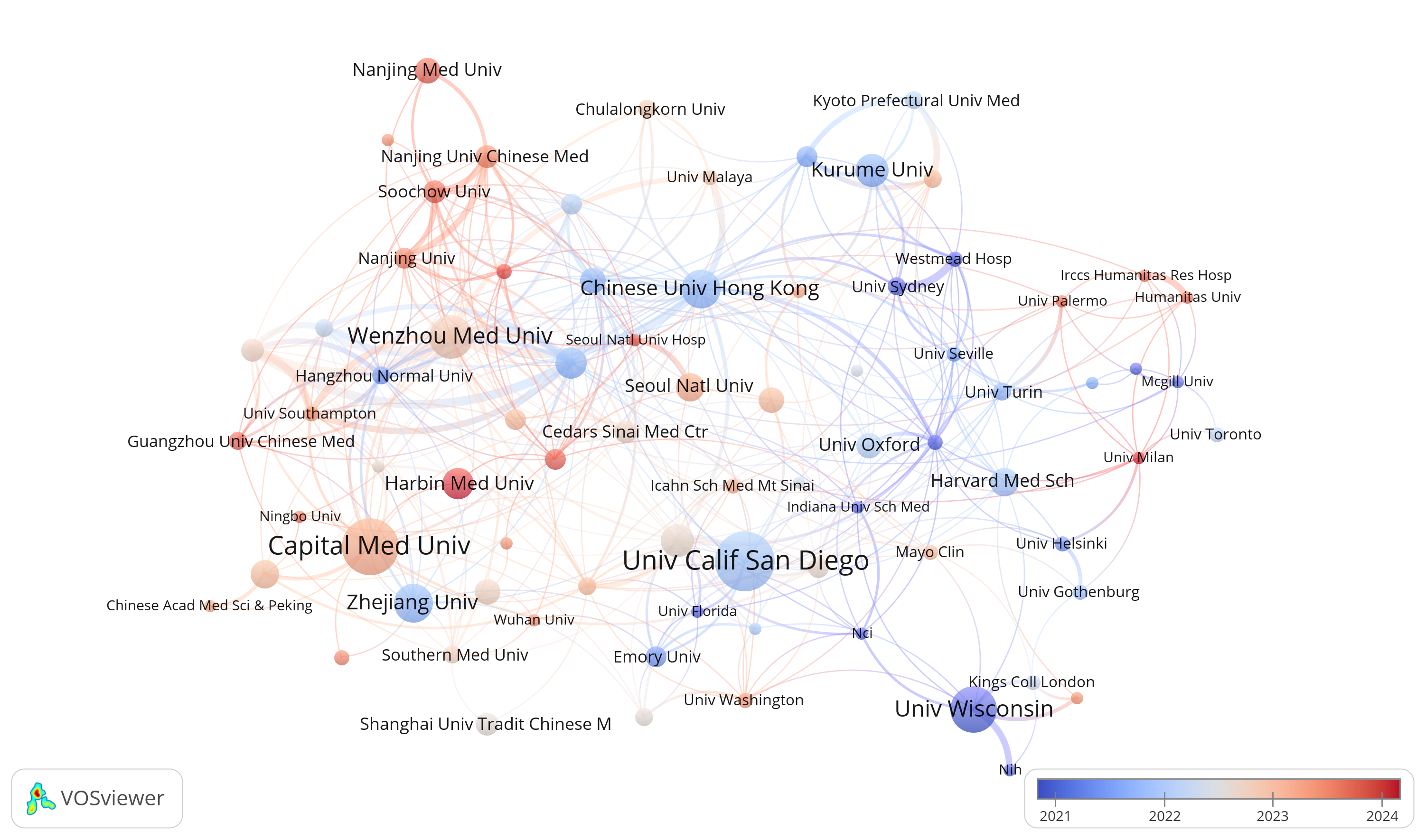

Supplement: Supplementary Figure 1 — Time Evolution of Institutional Collaboration Networks in Artificial Intelligence Research on Non-alcoholic Fatty Liver Disease from 2021-2024. Nodes represent research institutions, connecting lines represent collaborative relationships, and the color gradient from blue to red represents the temporal change from 2021 to 2024. This figure displays the institutional collaboration network for research related to non-alcoholic fatty liver disease (NAFLD) and artificial intelligence, presented in the form of a heat map. In the figure, each node represents a different research institution, while the lines between nodes indicate collaborative relationships between institutions; thicker lines represent stronger collaborative intensity. The color gradient from blue to red corresponds to the time span from 2021 to 2024, with red nodes indicating more active institutions in recent years (2023-2024), while blue nodes represent major participants in earlier years (2021-2022). [file Image1.jpeg]

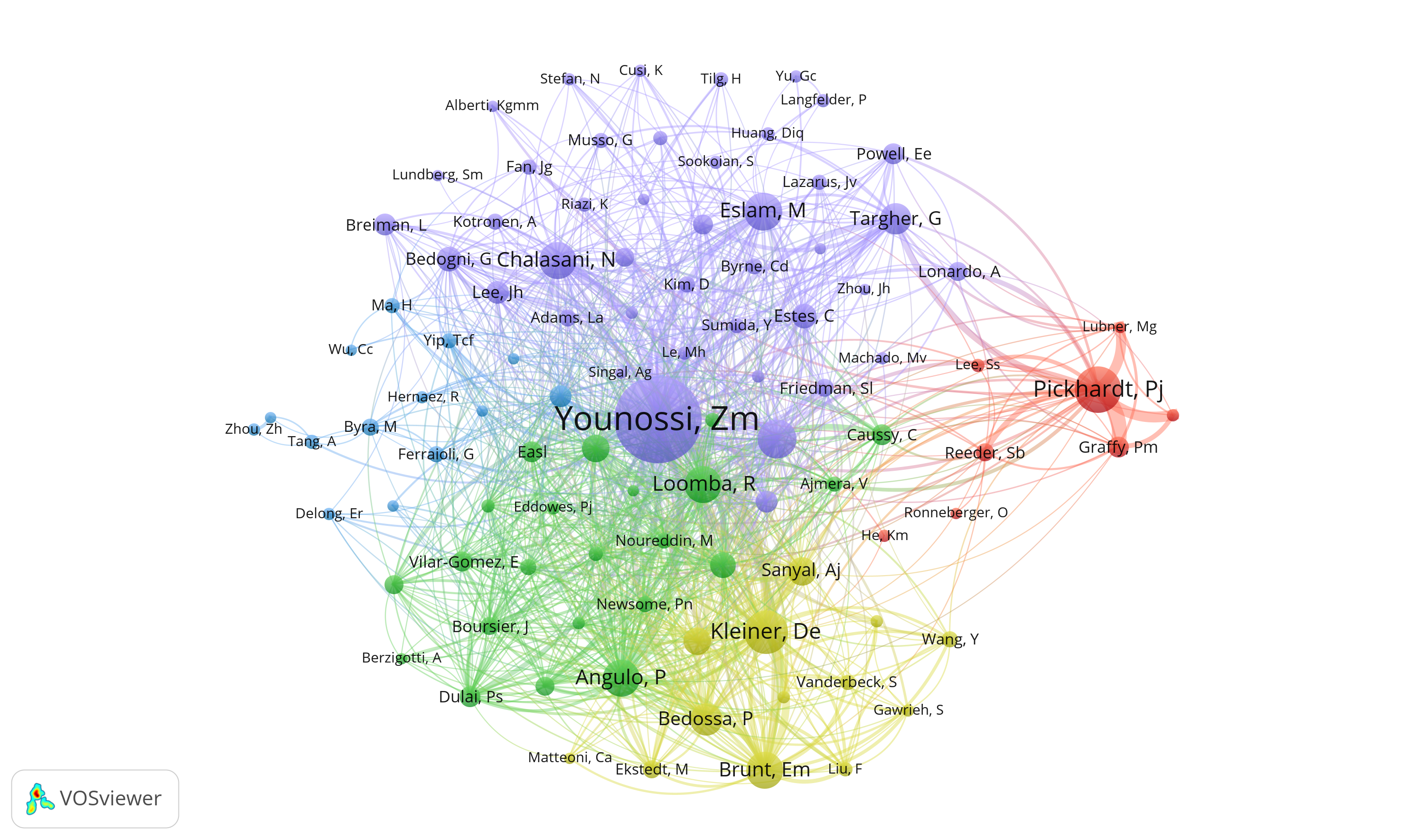

Supplement: Supplementary Figure 2 — Visualization of institutional collaboration network in the field of non-alcoholic fatty liver disease and artificial intelligence research (bright background). This network diagram shows collaborative relationships between research institutions, where nodes represent different institutions and connecting lines indicate collaborative relationships. Researchers such as Younossi ZM, Chalasani N, and others located in the central area occupy core positions in the network, indicating their significant influence in this field. Different colored clusters represent different research communities or directions. [file Image2.jpeg]

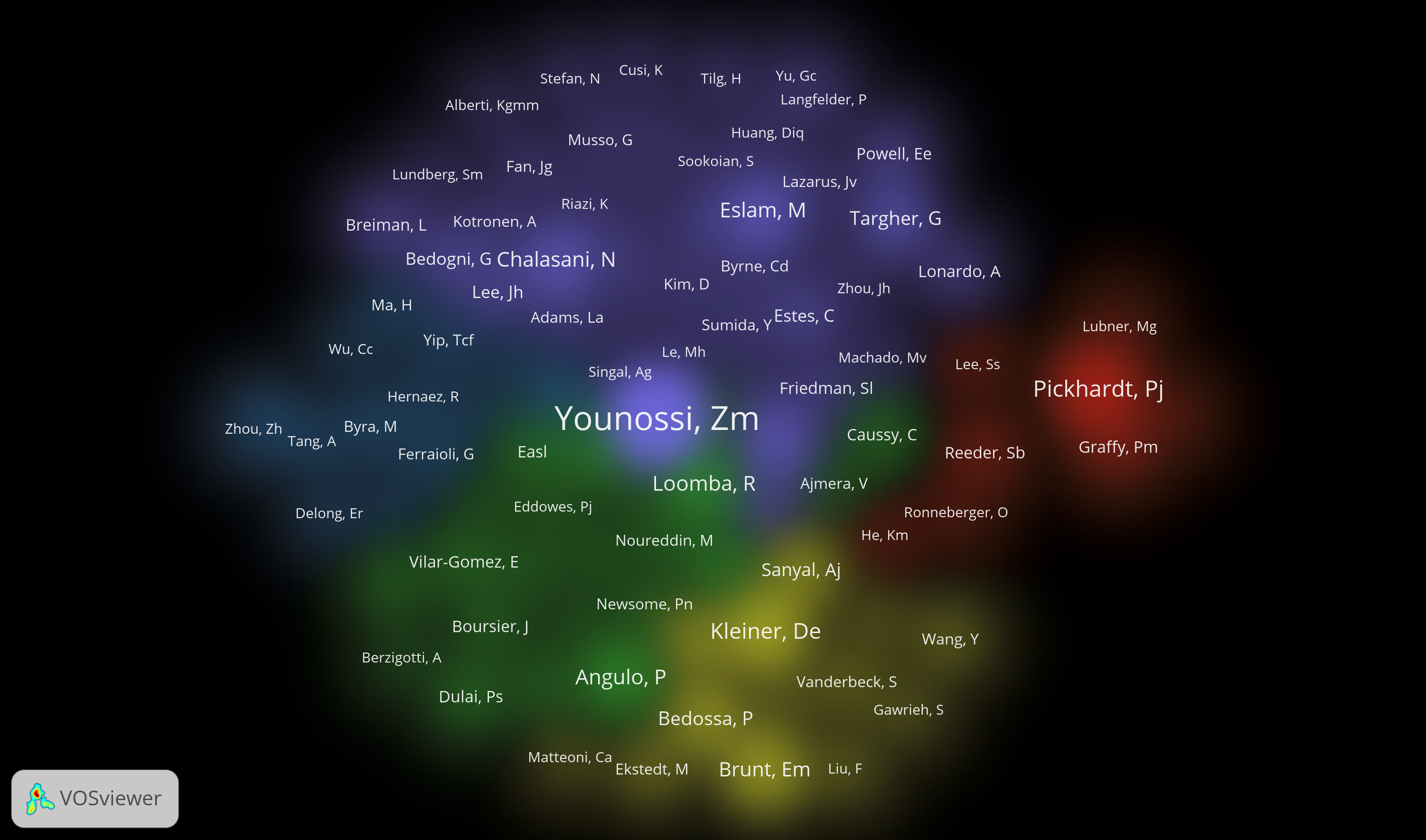

Supplement: Supplementary Figure 3 — Visualization of institutional collaboration network in the field of non-alcoholic fatty liver disease and artificial intelligence research (dark background). The diagram clearly shows the collaborative relationships between the research team centered around Younossi ZM and other research groups such as Chalasani N, Pickhardt PJ, etc. Different colors represent the distribution of different research communities. [file Image3.jpeg]

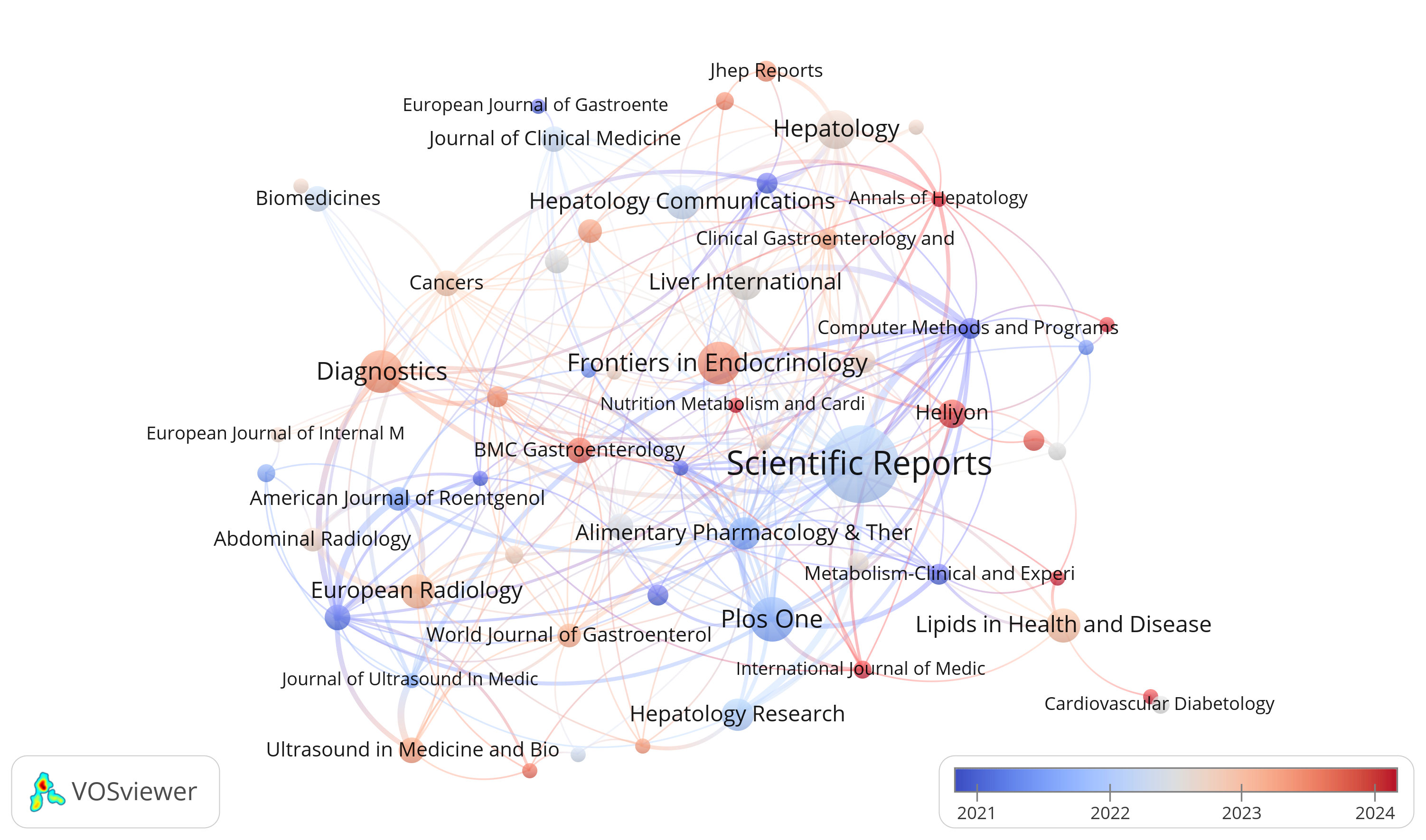

Supplement: Supplementary Figure 4 — Research Topic Distribution Map in the Field of Non-alcoholic Fatty Liver Disease and Artificial Intelligence Research. This network diagram illustrates the thematic distribution and interconnections within the research domains of non-alcoholic fatty liver disease and artificial intelligence. The nodes represent distinct research directions or keywords, while the connecting lines indicate the relationships among these topics. Multiple research clusters are distributed around the core area of “Scientific Reports,” encompassing hepatology, diagnostics, imaging, artificial intelligence applications, and related clinical fields. The color gradient from blue to red reflects the temporal evolution or research intensity changes of topics spanning from 2021 to 2024. [file Image4.jpeg]
